# Human tauopathy-derived tau strains determine the substrates recruited for templated amplification

**DOI:** 10.1093/brain/awab091

**Published:** 2021-03-09

**Authors:** Airi Tarutani, Haruka Miyata, Takashi Nonaka, Kazuko Hasegawa, Mari Yoshida, Yuko Saito, Shigeo Murayama, Andrew C Robinson, David M A Mann, Taisuke Tomita, Masato Hasegawa

**Affiliations:** 1 Department of Brain and Neurosciences, Tokyo Metropolitan Institute of Medical Science, Tokyo 156-8506, Japan; 2 Laboratory of Neuropathology and Neuroscience, Graduate School of Pharmaceutical Sciences, The University of Tokyo, Tokyo 113-0033, Japan; 3 Division of Neurology, Sagamihara National Hospital, Kanagawa 252-0392, Japan; 4 Department of Neuropathology, Institute for Medical Science of Aging, Aichi Medical University, Aichi 480-1195, Japan; 5 Department of Neuropathology, Tokyo Metropolitan Institute of Gerontology, Tokyo 173-0015, Japan; 6 Department of Pathology and Laboratory Medicine, National Center Hospital, National Center of Neurology and Psychiatry, Tokyo 187-8551, Japan; 7 Brain Bank for Neurodevelopmental, Neurological and Psychiatric Disorders, United Graduate School of Child Development, Osaka University, Osaka 565-0871, Japan; 8 Faculty of Biology, Medicine and Health, School of Biological Sciences, Division of Neuroscience and Experimental Psychology, The University of Manchester, Salford Royal Hospital, Salford M6 8HD, UK

**Keywords:** tau, prion-like propagation, tauopathy, strains

## Abstract

Tauopathies are a subset of neurodegenerative diseases characterized by abnormal tau inclusions. Specifically, three-repeat tau and four-repeat tau in Alzheimer’s disease, three-repeat tau in Pick’s disease (PiD) and four-repeat tau in progressive supranuclear palsy (PSP) and corticobasal degeneration (CBD) form amyloid-like fibrous structures that accumulate in neurons and/or glial cells. Amplification and cell-to-cell transmission of abnormal tau based on the prion hypothesis are believed to explain the onset and progression of tauopathies. Recent studies support not only the self-propagation of abnormal tau, but also the presence of conformationally distinct tau aggregates, namely tau strains. Cryogenic electron microscopy analyses of patient-derived tau filaments have revealed disease-specific ordered tau structures. However, it remains unclear whether the ultrastructural and biochemical properties of tau strains are inherited during the amplification of abnormal tau in the brain. In this study, we investigated template-dependent amplification of tau aggregates using a cellular model of seeded aggregation. Tau strains extracted from human tauopathies caused strain-dependent accumulation of insoluble filamentous tau in SH-SY5Y cells. The seeding activity towards full-length four-repeat tau substrate was highest in CBD-tau seeds, followed by PSP-tau and Alzheimer’s disease (AD)-tau seeds, while AD-tau seeds showed higher seeding activity than PiD-tau seeds towards three-repeat tau substrate. Abnormal tau amplified in cells inherited the ultrastructural and biochemical properties of the original seeds. These results strongly suggest that the structural differences of patient-derived tau strains underlie the diversity of tauopathies, and that seeded aggregation and filament formation mimicking the pathogenesis of sporadic tauopathy can be reproduced in cultured cells. Our results indicate that the disease-specific conformation of tau aggregates determines the tau isoform substrate that is recruited for templated amplification, and also influences the prion-like seeding activity.

## Teaser

Tarutani *et al*. show that pathogenic tau seeds extracted from the brains of patients with tauopathies induce disease-specific seeded tau aggregation and filament formation in cultured cells. The results indicate that template-dependent tau aggregation is key to the development of various types of tauopathy.

## Introduction

Abnormal proteins that accumulate in the brain of patients with neurodegenerative diseases form amyloid-like cross-β structures, and exhibit prion-like properties, including detergent-insolubility and protease resistance, which enable them to convert normal proteins to an abnormal form.^1^ Tau, which is one of these prion-like proteins, is a microtubule-associated protein encoded by the *MAPT* gene.^2^ In the adult human brain, tau exists in six isoforms composed of 352–441 residues with the different amino acid numbers arising from alternative splicing of exon 2, exons 2 and 3, or exon 10.^3^ These tau isoforms can be classified into three-repeat tau (3R tau) and four-repeat tau (4R tau) isoforms according to the number of repeats (R1–R4) in the microtubule-binding domain, and the two types are expressed in the adult human brain at a ratio of ∼1:1.^4^ Under physiological conditions, tau is localized mainly in axons as a soluble, natively unfolded protein, and functions to polymerize tubulins and stabilize microtubules.^5^ On the other hand, accumulated pathological tau in the brains of patients exists as filamentous insoluble forms that are hyperphosphorylated and partially ubiquitinated, and have lost the ability to bind to microtubules.^6^

Neurodegenerative diseases in which tau inclusions are observed in neurons and/or glial cells are referred to as tauopathies. In Alzheimer’s disease and chronic traumatic encephalopathy, six isoforms of tau accumulate as neurofibrillary tangles in neuronal cell bodies and neuropil threads in neurites.^7^ Astrocytic tangles are also a neuropathological feature in chronic traumatic encephalopathy.^8^ In Pick’s disease (PiD), 3R tau is observed in neurons as Pick bodies.^7^ In progressive supranuclear palsy (PSP) and corticobasal degeneration (CBD), 4R tau accumulates in neurons and glial cells.^9^ Although coiled bodies in oligodendrocytes are observed in both diseases, tufted astrocytes observed in PSP and astrocytic plaques observed in CBD are neuropathological indicators that enable each disease to be distinguished.^10^ Argyrophilic granular dementia and globular glial tauopathy are also categorized as 4R tauopathies.^9^ While most cases of tauopathy are sporadic, mutations in the *MAPT* gene cause the onset of familial frontotemporal dementia and parkinsonism linked to chromosome 17 (FTDP-17T).^11-14^ Multiple missense mutations have been found in and around the microtubule-binding domain, and affect the structure of tau and its binding to microtubules.^15^^,^^16^ In addition, mutations in exon 10 and intron 10 often alter the pattern of alternative splicing and lead to unequal ratios of 3R tau and 4R tau, resulting in the onset of tauopathy, depending on the increased tau isoform.

Histopathological studies indicate that the tau pathology observed in the brain of patients with Alzheimer’s disease and argyrophilic granular dementia correlates with clinical symptoms and spreads in the brain in a stereotypic manner.^17^^,^^18^ It has also been reported that PiD, PSP and CBD patients present with clinical symptoms related to the brain region in which tau pathology is observed.^19^ A possible mechanism to explain this intracerebral expansion of tau pathology is prion-like propagation, in which abnormal tau self-amplifies and spreads in the brain.^20^^,^^21^ Although the molecular mechanism of cell-to-cell transmission is poorly understood, numerous reports of experimental transmissions of abnormal tau *in vitro* and *in vivo* support this hypothesis.^22^^,^^23^ Recently, it was also proposed that the variety of clinical symptoms and pathologies in tauopathies can be explained by the involvement of distinct tau ‘strains’, which is a characteristic of prions. Biochemical analysis of patient-derived sarkosyl-insoluble tau shows that human tauopathies are characterized by distinct banding patterns of the C-terminal tau fragments (tau-CTFs) and trypsin-resistant tau.^24^^,^^25^ Moreover, tau filaments extracted from the brains of patients exhibit various ultrastructural properties depending on the tauopathy involved, and cryogenic electron microscopy (cryo-EM) of these tau filaments has revealed disease-specific ordered core structures at the atomic level.^26–28^ In experimental models of prion-like propagation, the physical and pathological properties of tau strains are inherited during the amplification of seeds and the formation of tau pathology.^29–34^ Thus, it has been suggested that the diversity of ultrastructural and biochemical properties of tau strains is the key to the formation of disease-specific tau pathology. However, the molecular mechanisms of formation of tau strains have not yet been elucidated, and it is not clear how the prion-like properties of tau strains are maintained during self-templated amplification and spreading in the brain.

In this study, we investigated template-dependent amplification of abnormal tau by introducing patient-derived tau strains into SH-SY5Y cells. Tau aggregates extracted from Alzheimer’s disease, PiD, PSP, and CBD cases caused isoform-dependent accumulation of insoluble filamentous tau, and these abnormal tau species showed ultrastructural and biochemical properties akin to those of the original seeds. Our results indicate that distinct conformations of patient-derived tau strains are involved in disease-specific tau filament formation.

## Materials and methods

### Ethics statement

Post-mortem brain tissues, which had been neuropathologically diagnosed as Huntington’s disease, Alzheimer’s disease, PiD, PSP, CBD and FTDP-17T, were obtained from the Brain Banks at the University of Manchester, Tokyo Metropolitan Geriatric Hospital and Institute of Gerontology, National Center of Neurology and Psychiatry, and Aichi Medical University. The study protocol was approved by the ethics committees of Tokyo Metropolitan Institute of Medical Science (18-9) and the University of Tokyo (30-7). All methods were performed in accordance with the relevant guidelines and regulations. All brain tissues used in this study were anonymized.

### Antibodies

Anti-tau antibodies used in this study were as follows: T46 (epitope: 404–441; Thermo Fisher Scientific), pS396 (epitope: p-Ser-396; Calbiochem), RD3 (epitope: 209–224; Millipore), Anti-4R (epitope: 275–291; Cosmo Bio), TauC (epitope: 429–441; Cosmo Bio), AT8 (epitope: p-Ser-202 and p-Thr-205; Thermo Fisher Scientific), tau 360–380 (epitope: 360–380; Cosmo Bio), pS262/pT263 (epitope: p-Ser-262 and p-Thr-263; abcam). Polyclonal anti-α-synuclein (α-syn) 131–140 antibody was obtained from Cosmo Bio. Monoclonal anti-α-tubulin antibody (T9028), monoclonal and polyclonal anti-HA antibodies (H3663 and H6908), and polyclonal anti-FLAG antibody (F7425) were obtained from Sigma.

### Preparation of sarkosyl-insoluble fractions from patients’ brains

Sarkosyl-insoluble fractions were prepared from patients’ brains as described^25^ and resuspended in saline by sonication for 15 s and used for electron microscopy or introduction into SH-SY5Y cells. For immunoblotting, sarkosyl-insoluble fractions were added to SDS-sample buffer and boiled for 3 min. The samples were separated on 4–20% gradient polyacrylamide gel (Wako) and immunoblotting was performed as described.^35^ For immunodepletion of tau from the sarkosyl-insoluble fraction, a mixture of AT8, tau 360–380 and TauC antibodies or anti-α-syn 131–140 antibody was coupled to Pierce^TM^ NHS-Activated Magnetic Beads (Thermo Scientific) according to the manufacturer’s instructions. The sarkosyl-insoluble fraction suspended in Tris-buffered saline with Tween-20 was incubated with tau antibody-coupled beads or α-syn antibody-coupled beads at 4°C overnight. The unbound sample was further incubated with new antibody-coupled beads as before. The resulting supernatant was collected as an immunodepleted sample and used for introduction into cells. α-Syn-depleted samples were used as a control. Trypsin treatment of the sarkosyl-insoluble fraction was performed as described.^25^ Trypsin was added at a final concentration of 0.1 mg/ml and incubated at 37°C for 30 min, and then the digestion was stopping by boiling.

### Quantification of total tau by ELISA

The concentrations of total tau in sarkosyl-insoluble fractions extracted from human brain samples were determined by sandwich ELISA. Wako human tau ELISA kit was purchased from FUJIFILM and used according to the manufacturer’s instructions.

### Electron microscopy

Sarkosyl-insoluble fractions extracted from Alzheimer’s disease, PiD, PSP, CBD and FTDP-17T patients’ brains were dropped onto carbon-coated 300-mesh copper grids (Nissin EM) and negatively stained as described.^36^ For immunostaining, sarkosyl-insoluble fractions extracted from transfected SH-SY5Y cells or patients’ brains were dropped onto grids and dried. The grids were immunostained with appropriate primary antibodies (1:50–100) and secondary antibodies conjugated to 10 nm gold particles (BBI Solutions, 1:50), 6 nm gold particles (abcam, 1:50) or 5 nm gold particles (Cytodiagnostics, 1:50) as described.^36^ Electron micrograph images were recorded with a JEOL JEM-1400 electron microscope.

### Cell culture, transfection of plasmids and introduction of pathogenic proteins into cells

Human neuroblastoma SH-SY5Y cells were maintained as described.^37^ Cells were cultured to 40–50% confluence in 6-well or 12-well plates and transfected with plasmids using X-tremeGENE^TM^ 9 (Roche Life Science) according to the manufacturer’s instructions. We used non-tagged human tau 3R1N, 4R1N, haemagglutinin (HA)-tagged human tau 3R1N, 4R1N, and FLAG-tagged human tau 3R1N, 4R1N in the pCDNA3.1 vector. After transfection of plasmids, cells were incubated for 6–8 h, and pathogenic tau seeds (2 μl for 6-well plate or 1–2μl for 12-well plate) were introduced using MultiFectam (Promega) according to the manufacturer’s instructions. Transfected cells were incubated for 3 days.

### Preparation of sarkosyl-insoluble fractions from transfected cells and immunoblotting

Transfected SH-SY5Y cells were collected and extracted with 1 ml for a 6-well plate or 0.5 ml for a 12-well plate of 1% sarkosyl in A68 buffer (10 mM Tris-HCl pH 7.5 containing 10% sucrose, 0.8 M NaCl, 1 mM EGTA). Cell extracts were sonicated for 15 s. After incubation for 30 min at 37°C, cell extracts were ultracentrifuged at 113 000*g* for 20 min at 25°C. The supernatants were removed and collected as sarkosyl-soluble fractions, then the pellets were washed with 30 mM Tris-HCl (pH 7.5) and ultracentrifuged as before. The resulting pellets were collected as sarkosyl-insoluble fractions, resuspended in 30 mM Tris-HCl (pH 7.5) and sonicated for 15 s. These fractions were used for immunoelectron microscopy and as seeds for multiple passages of insoluble tau in SH-SY5Y cells. Sarkosyl-insoluble and soluble fractions were added to SDS-sample buffer and boiled for 3 min, and immunoblotting was performed. Monoclonal anti-α-tubulin was used to obtain a loading control. The protein concentrations of samples were determined with a Pierce BCA Protein Assay Kit (Thermo Fisher Scientific). All experiments were performed at least three times. The band intensities of immunoblots were quantified using ImageQuant TL (cytiva) and were analysed using Prism software (GraphPad Software).

### Confocal immunofluorescence microscopy

Transfection of plasmids and introduction of pathogenic tau seeds were conducted as described above, using SH-SY5Y cells grown on coverslips. After incubation for 3 days, cells were fixed with 4% paraformaldehyde and treated with AT8 and TauC antibodies and the secondary antibodies (anti-mouse IgG-conjugated Alexa-488 and anti-rabbit IgG-conjugated Alexa-568, Invitrogen) as described.^37^ The cells were mounted and analysed using a LSM780 confocal laser microscope (Carl Zeiss).

### Statistical analysis

Welch’s modified *t*-test was performed for Fig. 2B. Unpaired *t*-test was carried out for SupplementaryFig.3DandG. In all cases, a *P*-value < 0.05 was regarded as statistically significant. Statistical analyses were performed using Prism software (GraphPad Software).

### Data availability

All raw data used for figure generation in this study can be obtained by contacting the corresponding author.

## Results

### Biochemical and ultrastructural characterization of patient-derived tau aggregates

**Figure 1:**
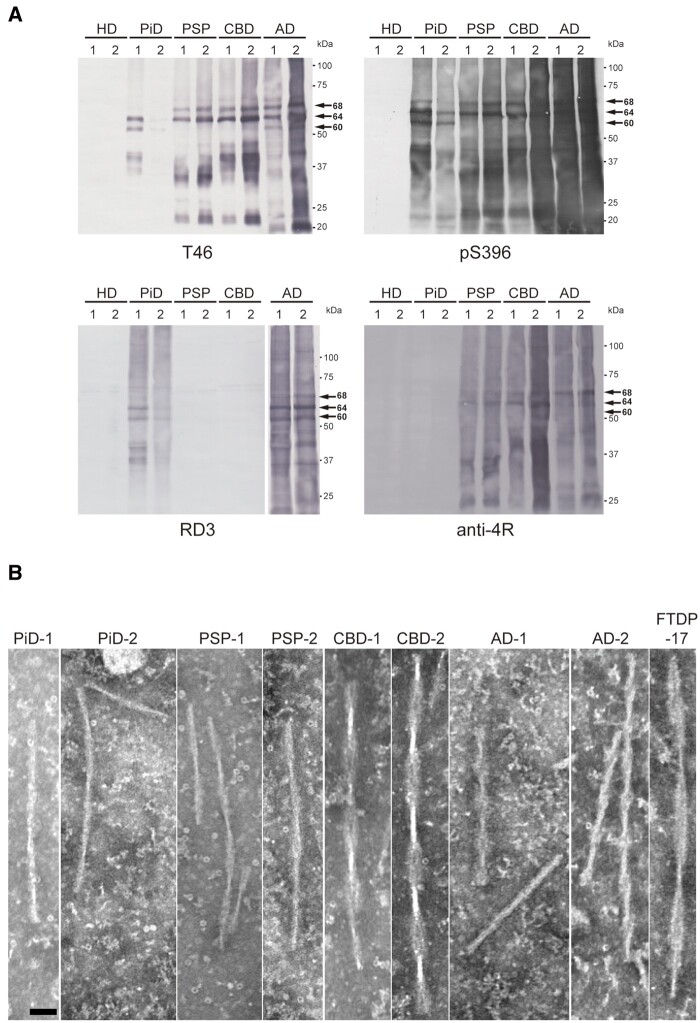
**Biochemical and ultrastructural characterization of abnormal tau extracted from human tauopathies**. (**A**) Immunoblot analyses of sarkosyl-insoluble fractions prepared from brains of tauopathy and Huntington’s disease patients. Sarkosyl-insoluble full-length tau (60, 64 and 68 kDa) and CTFs were detected with T46 (*top* *left*) and pS396 (*top* *right*) antibodies. 3R tau and 4R tau were detected with RD3 (*bottom* *left*) and anti-4R (*bottom* *left*) antibodies, respectively. Full-length blots are presented in the Supplementary material. (**B**) Electron microscopy of sarkosyl-insoluble fractions extracted from PiD, PSP, CBD, Alzheimer’s disease and FTDP-17T cases. Scale bar = 50 nm.

First, we prepared the sarkosyl-insoluble fractions from the brains of patients neuropathologically diagnosed as Alzheimer’s disease, PiD, PSP and CBD, and investigated the biochemical and ultrastructural properties of tau aggregates contained in these fractions (Supplementary Table 1). Immunoblot analysis of sarkosyl-insoluble fractions with T46, which recognizes the C-terminal tau (404–441), and pS396 tau antibodies showed that different tau isoforms accumulated in each tauopathy: there were two major bands at 60 and 64 kDa in PiD, two major bands at 64 and 68 kDa in PSP and CBD, and three major bands at 60, 64 and 68 kDa in Alzheimer’s disease, which represent hyperphosphorylated full-length tau proteins (Fig. 1A and Supplementary Fig. 1). As reported previously,^24^^,^^25^ disease-specific tau-CTFs, 21, 34 and 39 kDa tau in PiD, 22 and 33 kDa tau in PSP, 22, 37–40 and 43 kDa tau in CBD and 19, 22, 25, 30, 36 and 40 kDa tau in Alzheimer’s disease were also detected (Fig. 1A and Supplementary Fig. 1). We also confirmed the identities of the tau isoforms accumulated in each case using tau isoform-specific antibodies, RD3 and anti-4R (Fig. 1A and Supplementary Fig. 1).^38^ Abnormal tau was not detected in the sarkosyl-insoluble fractions extracted from two cases with Huntington’s disease, and these cases were used as a control not containing abnormal tau in subsequent experiments (Fig. 1A).

Furthermore, the sarkosyl-insoluble fractions were negatively stained and observed by electron microscopy (Fig. 1B). In PiD cases, straight filaments with 13–17 nm diameter and twisted filaments with 129.8 [±5.0, mean standard deviation (SD)] nm periodicity were observed. In PSP cases, thin filaments with wide regions of 14.9 (±1.7) nm diameter and narrow regions of 7 nm diameter were loosely twisted with 108.7 (±4.5) nm periodicity. Tau aggregates derived from CBD cases are characterized by wider filaments than those in PSP, and CBD filaments with wide regions of 28.9 (±3.2) nm diameter and narrow regions of 8.5 nm diameter were twisted with 138.9 (±11.7) nm periodicity. Ribbon-like filaments with wide regions of 25.2 (±6.7) nm diameter and narrow regions of 7 nm diameter were twisted with 236.5 (±21.2) nm periodicity in the case of FTDP-17T with intron 10 mutation +16, which increases the expression of 4R tau, indicating the diversity of tau filament structures within 4R tauopathy. In Alzheimer’s disease cases, straight filaments with 14.4 (±1.6) nm diameter and paired helical filaments (PHFs) with wide regions of 19.3 (±2.1) nm diameter and narrow regions of 10 nm diameter, which were twisted with 81.7 (±6.6) nm periodicity, were observed. These ultrastructural properties of tau aggregates derived from human tauopathies are consistent with previous electron microscopy observations of patient-derived tau filaments.^26^^,^^27^ These results indicate that tau aggregates extracted from the Alzheimer’s disease, PiD, PSP, and CBD cases used in this study are distinct tau strains having different biochemical and ultrastructural properties.

### Seeded tau aggregation induced by patient-derived tau strains in SH-SY5Y cells

**Figure 2:**
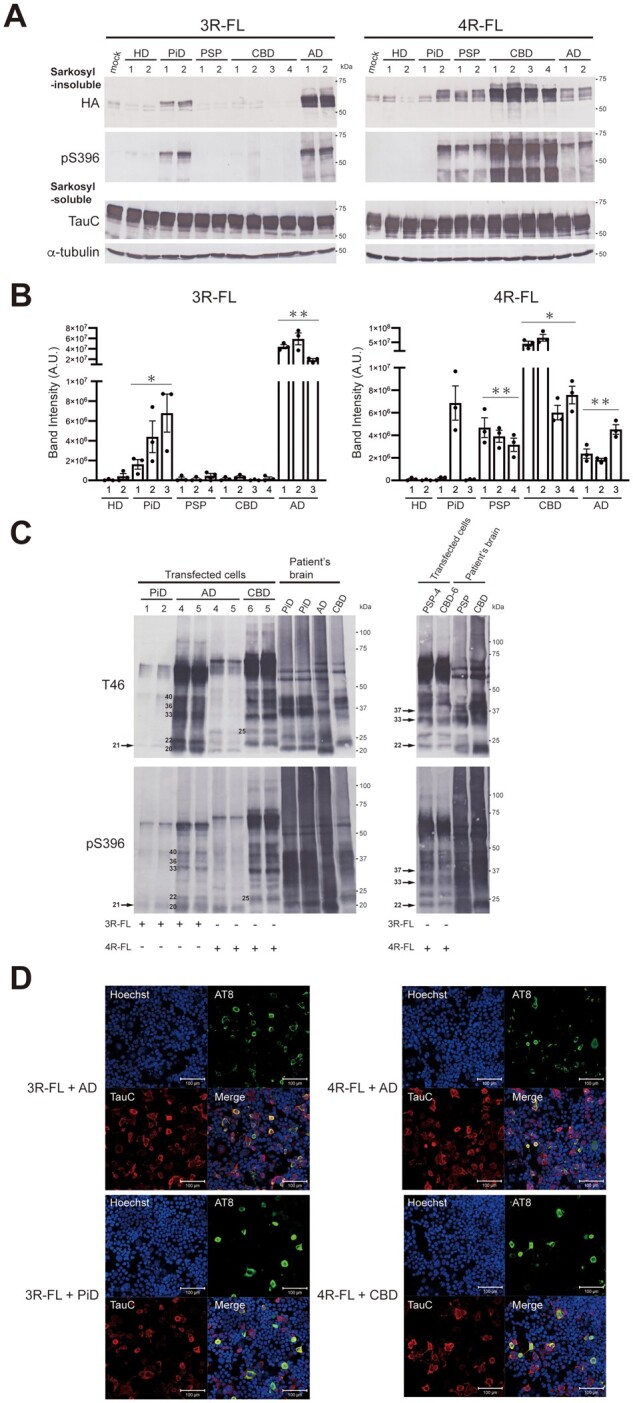
**Seeded tau aggregation induced by patient-derived tau strains in SH-SY5Y cells expressing full-length tau.** (**A**) Sarkosyl-insoluble fractions extracted from patients’ brains (1 μl) were introduced into SH-SY5Y cells transiently expressing HA-tagged wild-type human tau 3R1N (*left*) or 4R1N (*right*). Immunoblot analysis of sarkosyl-insoluble fractions and sarkosyl-soluble fractions extracted from mock-transfected cells, and cells transfected with sarkosyl-insoluble fractions from two Huntington’s disease cases, two PiD cases, two PSP cases, four CBD cases and two Alzheimer’s disease cases. Insoluble tau was detected with anti-HA and pS396 antibodies. Total tau was detected with TauC antibody. The tau concentrations of sarkosyl-insoluble fractions derived from human brains are shown in Supplementary Table 2. Full-length blots are presented in the Supplementary material. (**B**) Quantification of the band intensities of the immunoblots with anti-HA antibody shown in **A** and Supplementary Fig. 2B. The results are expressed as means ± standard error of the mean (SEM) (*n* = 3). *P < 0.01; **P < 0.001; Welch’s modified *t*-test against the value of Huntington’s disease. (**C**) Immunoblot analyses of sarkosyl-insoluble fractions prepared from transfected SH-SY5Y cells and patients’ brains. C-Terminal tau fragments were detected with T46 (*top*) and pS396 (*bottom*) antibodies. Full-length blots are presented in the Supplementary material. (**D**) SH-SY5Y cells introduced with AD-, PiD- and CBD-tau seeds were fixed and immunostained with AT8 (green) and TauC (red) antibodies. Scale bar = 100 μm.

**Figure 3:**
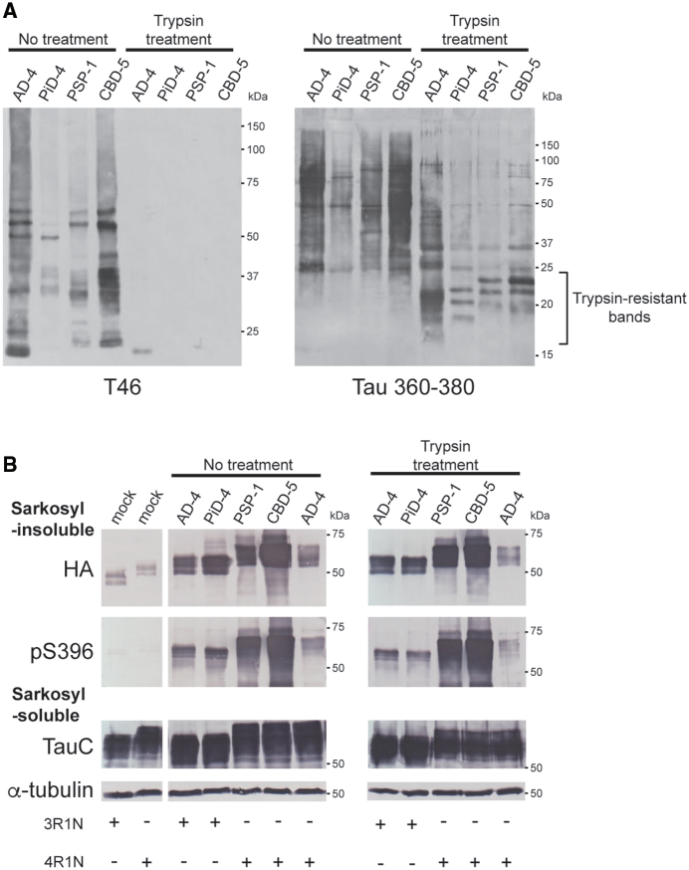
**Prion-like seeding activities of trypsin-treated insoluble fractions of tauopathy cases in SH-SY5Y cells.** (**A**) Immunoblot analyses of the sarkosyl-insoluble fractions before and after trypsin treatment. Full-length tau and C-terminal tau fragments were detected with T46 antibody. Trypsin-resistant tau bands were detected with tau 360–380 antibody. Full-length blots are presented in the Supplementary material. (**B**) The untreated and trypsin-treated insoluble fractions were introduced into SH-SY5Y cells transiently expressing HA-tagged human tau 3R1N or 4R1N. Immunoblot analyses of sarkosyl-insoluble fractions and sarkosyl-soluble fractions extracted from mock-transfected cells, and cells with introduced untreated or trypsin-treated tau seeds. Insoluble tau was detected with anti-HA and pS396 antibodies. Total tau was detected with TauC antibody. Full-length blots are presented in the Supplementary material.

To characterize the prion-like properties of tau strains derived from human tauopathies, we investigated cellular seeded tau aggregation induced by the introduction of pathogenic tau seeds. The sarkosyl-insoluble fractions derived from Alzheimer’s disease, PiD, PSP, CBD and Huntington’s disease patients’ brains were introduced into SH-SY5Y cells transiently expressing HA-tagged human full-length 3R tau (3R-FL) and 4R tau (4R-FL) without pathogenic mutations. We used the 1N isoform with exon 2 inserted, which shows the highest expression level in the human adult brain.^39^ Three days after the introduction of the seeds, the sarkosyl-insoluble fractions were extracted from transfected cells, and the accumulation of insoluble tau was evaluated by immunoblotting using anti-HA and pS396 antibodies (Fig. 2A and B). Introduction of pathogenic tau seeds into SH-SY5Y cells without transient expression of tau did not induce seeded accumulation of insoluble tau, and the added seeds themselves were also not detected (Supplementary Fig. 2A). Alzheimer’s disease, PiD, PSP and CBD-derived tau seeds caused strain-dependent seeded tau aggregation in SH-SY5Y cells (Fig. 2A, B and Supplementary Fig. 2B). PiD-tau seeds induced seeded aggregation of 3R tau in cells expressing 3R-FL, while PSP- and CBD-tau seeds caused seeded aggregation of 4R tau only in cells expressing 4R-FL. Insoluble tau accumulated in cells expressing 3R-FL or 4R-FL after introduction of Alzheimer’s disease (AD)-tau seeds. Huntington’s disease seeds did not cause seeded aggregation of either 3R-FL or 4R-FL. Such isoform-specific seeded tau aggregation induced by tau from human tauopathies was also observed in SH-SY5Y cells expressing non-tagged human full-length tau (Supplementary Fig. 2C). Furthermore, the amount of accumulated insoluble full-length tau generated by the addition of patient-derived tau seeds varied depending on the strain. The seeding activity of AD-tau seeds for 3R-FL was higher than that of PiD-tau seeds (Fig. 2B). In addition, CBD-tau seeds tended to exhibit higher seeding activity towards 4R-FL as compared to PSP- and AD-tau seeds (Fig. 2B). These differences were independent of the tau concentration in seeds (Supplementary Table 2). Unexpectedly, PiD-tau seeds from the PiD-2 case caused isoform-independent tau aggregation. Although immunoblotting with anti-4R (Fig. 1A) did not detect 4R tau in the PiD-2 case, PiD-tau seeds derived from this case induced the accumulation of insoluble tau not only in cells expressing 3R-FL, but also in cells expressing 4R-FL (Fig. 2A and B). To investigate whether this isoform-independent tau aggregation was caused by non-tau contamination in the sarkosyl-insoluble fraction, we further purified this fraction. CBB staining of purified samples obtained by resuspension and additional ultracentrifugation showed a decrease in the α- and β-tubulin bands at ∼50 kDa, one of the major contaminants in the insoluble fraction extracted from human brains (Supplementary Fig. 3A).^40^ Therefore, the unpurified PiD-1 and PiD-2 seeds or the purified PiD-2 seeds, whose total tau concentration was adjusted to 2 ng/μl, were introduced into cells expressing HA-tagged 3R-FL or 4R-FL and accumulation of insoluble tau was evaluated. Although the same amount of abnormal tau was introduced, the addition of the purified PiD-2 seeds significantly reduced the accumulation of 4R tau compared to the unpurified PiD-2 seeds (Supplementary Fig. 3B–D). These results suggest that the accumulation of insoluble 4R tau induced by the unpurified PiD-2 seeds was caused by some brain-derived contaminant(s), not by cross-seeding. Furthermore, tau-depleted samples from the sarkosyl-insoluble fraction were introduced into cells expressing HA-tagged 3R-FL or 4R-FL (Supplementary Fig. 3E–G). Accumulation of insoluble tau in cells introduced with tau-depleted samples was significantly reduced compared to cells introduced with control samples, suggesting that insoluble tau contained in the sarkosyl-insoluble fraction induced tau aggregation (Supplementary Fig. 3F and G). In addition, the C-terminal banding pattern of insoluble tau extracted from transfected cells was compared with that of patient-derived insoluble fractions used as seeds (Fig. 2C). Immunoblotting with T46 and pS396 antibodies of sarkosyl-insoluble fractions extracted from transfected cells detected CTFs of 21 kDa in cells with introduced PiD-tau seeds and 22, 33 and 37 kDa in cells with PSP- and CBD-tau seeds. Although these CTFs resembled patient-derived CTFs, CTFs derived from transfected cells with introduced PSP- and CBD-tau seeds were not clearly distinguishable. In the case of introduction of AD-tau seeds, CTFs of 20, 22, 30, 36, 40 kDa were detected in insoluble fractions extracted from cells expressing 3R tau, and CTFs of 20, 22, 25, 36, 40 kDa were detected in insoluble fractions extracted from cells expressing 4R tau. Tau-CTFs resembling patient-derived tau-CTFs were also detected in insoluble tau accumulated in transfected cells expressing non-tagged 3R-FL or 4R-FL (Supplementary Fig. 2D). These results indicate that the abnormal tau amplified in SH-SY5Y cells inherits the ultrastructural properties of patient-derived tau seeds. Immunohistological staining with AT8, which is the most frequently used antibody in the pathological diagnosis of tauopathies, showed that tau aggregates were formed in TauC-positive cells with introduced patient-derived tau seeds, consistent with the accumulation of insoluble tau detected by immunoblotting (Fig. 2D). On the other hand, AT8-positive tau was not detected in cells without transient expression of tau, mock cells co-expressing both 3R tau and 4R tau, or transfected cells without transient expression of tau with introduced AD-tau seeds (Supplementary Fig. 4). Thus, the sarkosyl-insoluble fractions extracted from the brains of patients with tauopathies caused strain-dependent seeded aggregation of full-length tau substrates, except for the insoluble fraction derived from the PiD-2 case. These results indicate that disease-specific amplification of tau seeds that occurs in the brains of patients with tauopathies was reproduced in cultured cells, and distinct patient-derived strains exhibited a diversity of prion-like properties, including isoform specificity and seeding activity. To investigate the effect of N-terminal and C-terminal tau regions (fuzzy coat) that are not a part of the fibrous core on seeded aggregation, the sarkosyl-insoluble fractions extracted from the patient’s brain were treated with trypsin and the resulting trypsinized tau seeds were introduced into cells (Fig. 3). The trypsinized tau seeds induced seeded aggregation similarly to non-trypsinized tau seeds, indicating that the trypsin-resistant fibrous core contributes to the prion-like properties (Fig. 3B).^25^

### Isoform-specific seeded tau aggregation in the presence of both 3R tau and 4R tau substrates

**Figure 4:**
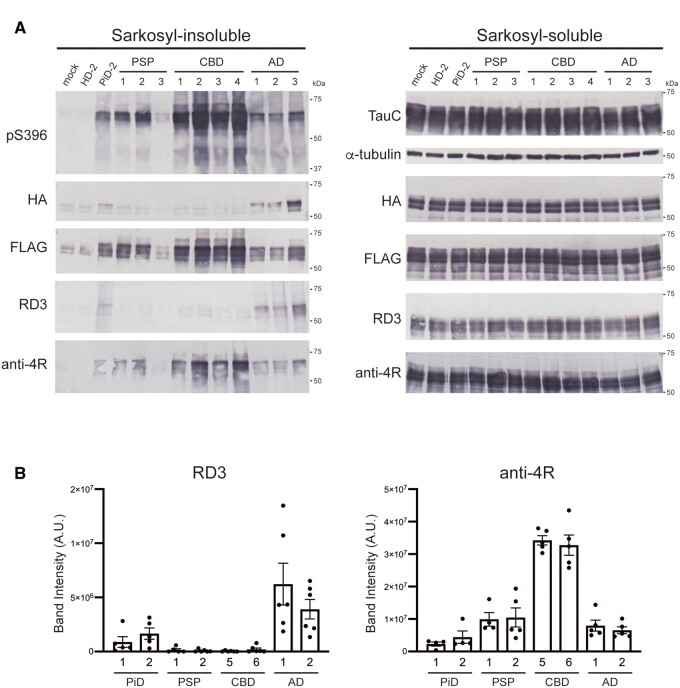
**Seeded tau aggregation induced by patient-derived tau strains in SH-SY5Y cells co-expressing 3R tau and 4R tau.** (**A**) Sarkosyl-insoluble fractions extracted from patients’ brains (1 μl) were introduced into SH-SY5Y cells transiently co-expressing HA-tagged human tau 3R1N and FLAG-tagged human tau 4R1N. Immunoblot analysis of sarkosyl-insoluble fractions (*left*) and sarkosyl-soluble fractions (*right*) extracted from mock-transfected cells, and cells with introduced sarkosyl-insoluble fractions from a Huntington’s disease case, a PiD case, three PSP cases, four CBD cases and three Alzheimer’s disease cases. Insoluble tau was detected with pS396, anti-HA, anti-FLAG, RD3 and anti-4R antibodies. Total tau was detected with TauC, anti-HA, anti-FLAG, RD3 and anti-4R antibodies. The tau concentrations of pathogenic tau seeds derived from human brains are shown in Supplementary Table 2. Full-length blots are presented in the Supplementary material. (**B**) Quantification of insoluble tau accumulated in transfected SH-SY5Y cells co-expressing human tau 3R1N and 4R1N with patient-derived tau seeds. The band intensities of the immunoblots with RD3 (*left*) and anti-4R (*right*) antibodies shown in Supplementary Fig. 5 were evaluated. The results are expressed as means ± SEM (*n* = 4–6).

We next investigated whether strain-dependent seeded tau aggregation induced by the introduction of patient-derived tau seeds occurs in the presence of both 3R tau and 4R tau substrates. Pathogenic tau seeds were introduced into SH-SY5Y cells co-expressing HA-tagged 3R-FL and FLAG-tagged 4R-FL, and seeded tau aggregation was evaluated (Fig. 4). Accumulation of insoluble 3R tau detected with anti-HA and RD3 antibodies was observed in cells with introduced PiD- and AD-tau seeds. Insoluble 4R tau was detected with anti-FLAG and anti-4R antibodies in cells with introduced PSP-, CBD- and AD-tau seeds (Fig. 4A). Evaluation of the insoluble fractions extracted from transfected cells with RD3 and anti-4R antibodies also showed that the tau seeds derived from human tauopathies caused isoform-specific seeded tau aggregation in the presence of both 3R tau and 4R tau substrates, except for the accumulation of insoluble 4R tau induced by the unpurified PiD-2 seeds (Fig. 4B and Supplementary Fig. 5). These results indicate that pathogenic tau seeds recruit tau isoforms for seeded aggregation in a substrate-selective manner, that is, the conformations of patient-derived tau strains determine which tau isoforms are selectively accumulated.

### Evaluation of prion-like seeding activities of patient-derived tau strains

**Figure 5:**
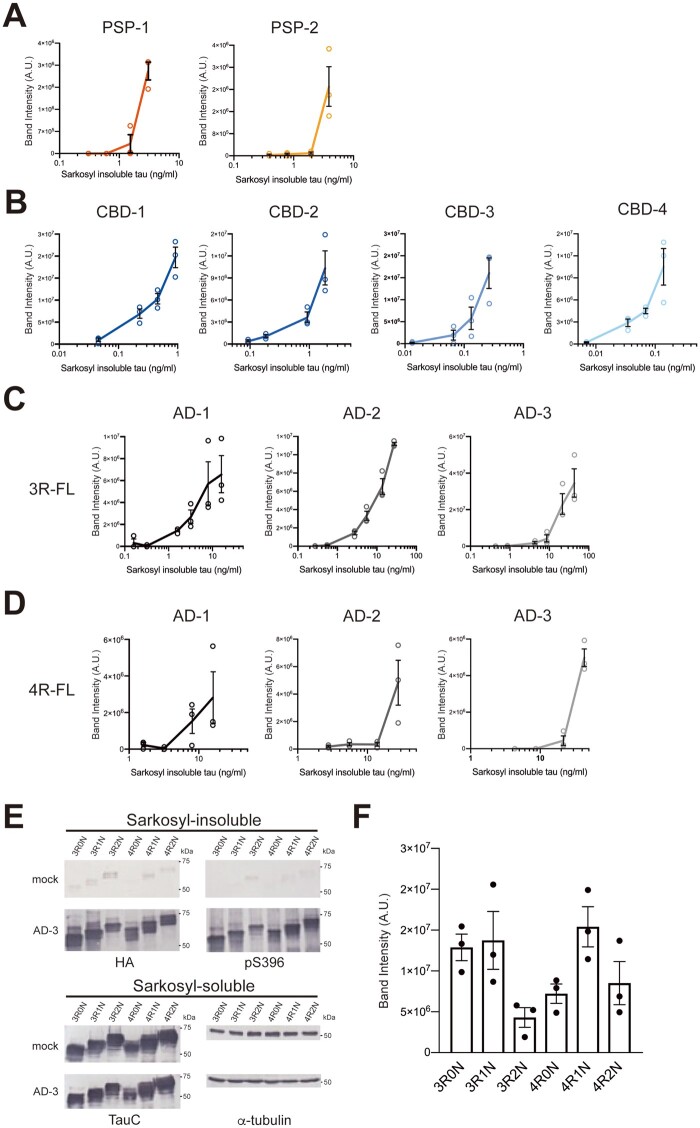
**Evaluation of prion-like seeding properties of tau aggregates derived from PSP, CBD and Alzheimer’s disease cases in SH-SY5Y cells.** (**A**) Serial dilutions of sarkosyl-insoluble fractions prepared from 2 PSP cases (1 μl) were introduced into SH-SY5Y cells transiently expressing HA-tagged human tau 4R1N. The band intensities of the immunoblots with anti-HA antibody shown in Supplementary Fig. 6A were evaluated. (**B**) Serial dilutions of sarkosyl-insoluble fractions prepared from four CBD cases (1 μl) were introduced into SH-SY5Y cells transiently expressing HA-tagged human tau 4R1N. The band intensities of the immunoblots with anti-HA antibody shown in Supplementary Fig. 6B were evaluated. The results are expressed as means ± SEM (*n* = 3). (**C**) Serial dilutions of sarkosyl-insoluble fractions prepared from three Alzheimer’s disease cases (1 μl) were introduced into SH-SY5Y cells transiently expressing HA-tagged human tau 3R1N. The band intensities of the immunoblots with anti-HA antibody shown in Supplementary Fig. 6C were quantified. (**D**) Serial dilutions of sarkosyl-insoluble fractions prepared from three Alzheimer’s disease cases (1 μl) were introduced into SH-SY5Y cells transiently expressing HA-tagged human tau 4R1N. The band intensities of the immunoblots shown in Supplementary Fig. 6D were evaluated. (**E**) Sarkosyl-insoluble fractions prepared from the Case AD-3 (1 μl) were introduced into SH-SY5Y cells transiently expressing HA-tagged human tau 3R0N, 3R1N, 3R2N, 4R0N, 4R1N and 4R2N, respectively. Immunoblot analysis of sarkosyl-insoluble fractions (*top*) and sarkosyl-soluble fractions (*bottom*) extracted from mock-transfected cells, and cells introduced with sarkosyl-insoluble fractions from Case AD-3. Insoluble tau was detected with pS396 and anti-HA antibodies. Total tau was detected with TauC antibody. Full-length blots are presented in the Supplementary material. (**F**) Quantification of the band intensities of the immunoblots with anti-HA antibody shown in **E**. The results are expressed as means ± SEM (*n* = 3).

Further, we examined whether pathogenic tau seeds derived from human tauopathies exhibit the seeding activities that characterize the tau strains. Total tau concentration contained in the sarkosyl-insoluble fraction was determined by sandwich ELISA using antibody that recognizes C-terminal tau as a capture antibody. Serial dilutions of PSP- and CBD-tau seeds were introduced into SH-SY5Y cells expressing HA-tagged 4R-FL, and the seeding activity was evaluated (Fig. 5A, B and Supplementary Fig. 6A and B). PSP-tau seeds derived from PSP-1 and PSP-2 cases showed high seeding activity at total tau concentrations in the seeds of 3.8 ng/ml and 5.8 ng/ml, respectively, but the seeds had almost no seeding activity at the tau concentration of 1 ng/ml or less (Fig. 5A and Supplementary Fig. 6A). On the other hand, CBD-tau seeds derived from Cases CBD-1–4 showed high seeding activity at 1 ng/ml or higher concentration, while the seeding activity decreased at 0.1 ng/ml or lower concentration (Fig. 5B and Supplementary Fig. 6B). CBD-tau seeds derived from the CBD-4 case showed the highest seeding activity, being effective even at 0.1 ng/ml, while the activity decreased at 0.04 ng/ml or less (Fig. 5B). Thus, tau seeds derived from PSP and CBD cases vary by 10- to 100-fold in their prion-like seeding activity. Furthermore, we prepared serial dilutions of AD-tau seeds and introduced them into SH-SY5Y cells expressing HA-tagged 3R-FL or 4R-FL (Fig. 5C, D and Supplementary Fig. 6C and D). In the presence of 3R tau substrate, AD-tau seeds derived from Cases AD-1–3 showed high seeding activity at 1.6 ng/ml, 2.76 ng/ml and 8.62 ng/ml, respectively, while the seeding activity was completely lost at 0.32 ng/ml, 0.55 ng/ml and 4.31 ng/ml, respectively (Fig. 5C and Supplementary Fig. 6C). In the presence of 4R tau substrate, AD-tau seeds derived from the Cases AD-1–3 showed high seeding activity at 19.2 ng/ml, 27.57 ng/ml and 43.11 ng/ml or higher, respectively, and the seeding activity was lost below 3.84 ng/ml, 13.78 ng/ml and 8.6 ng/ml, respectively (Fig. 5D and Supplementary Fig. 6D). Prion-like seeding activity of AD-tau seeds towards 3R-FL was more than 10 times higher than that towards 4R-FL in the three Alzheimer’s disease cases and consequently the seeding activity of AD-tau seeds towards 4R tau substrate was lower than that of PSP-tau seeds. For PiD-tau seeds, the total tau concentrations used in this study are the detection limits for induction of seeded aggregation, and the amount of insoluble tau accumulated in cells was much less than that observed in cells with introduced AD-tau seeds of the same tau concentration (Figs 2A, B and 5C). Furthermore, addition of AD-tau seeds derived from the AD-3 case to SH-SY5Y cells expressing each of six human tau isoforms showed that AD-tau seeds have the ability to induce seeded aggregation of all tau isoforms (Fig. 5E and F). Similarly, we confirmed that PiD-tau seeds derived from the PiD-2 case and CBD-tau seeds derived from the CBD-4 case induced seeded aggregation of the three 3R tau isoforms and three 4R tau isoforms, respectively (Supplementary Fig. 7AandB). These results suggest that different conformations of patient-derived tau strains may exert distinct prion-like seeding activities. In addition, although the accumulation ratio of insoluble 3R tau and 4R tau induced by AD-tau seeds was almost the same in the presence of both 3R tau and 4R tau (Fig. 4A and Supplementary Fig. 8B), AD-tau seeds exhibited higher seeding activity in the presence of only 3R tau substrate than in the presence of only 4R tau substrate.

### Template-dependent tau filament formation in transfected cells with patient-derived tau strains

**Figure 6:**
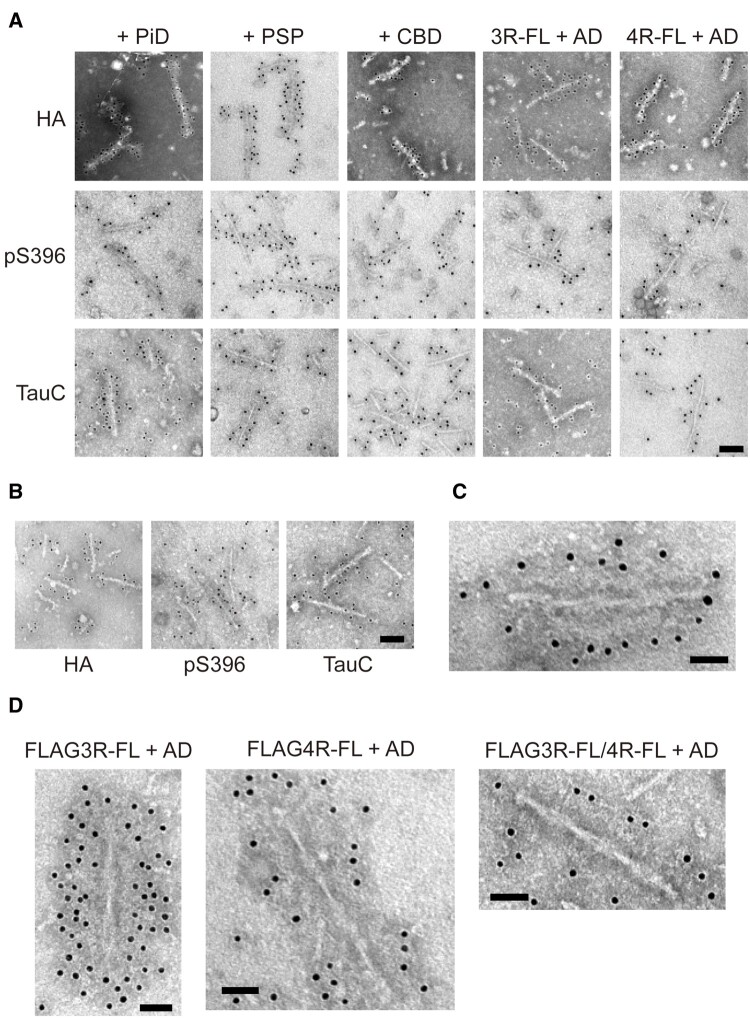
**Electron microscopic analysis of insoluble fractions extracted from SH-SY5Y cells transfected with patient-derived tau strains.** (**A**) Immunoelectron microscopy of sarkosyl-insoluble fractions extracted from transfected cells expressing HA-tagged human tau 3R1N or 4R1N with PiD-, PSP-, CBD- and AD-tau seeds. Electron micrographs show fibrous structures positive for anti-HA (*top*), pS396 (*middle*) and TauC (*bottom*) antibodies, that were labelled with secondary antibody conjugated to 10 nm gold particles. Scale bar = 100 nm. (**B**) Immunoelectron microscopy of sarkosyl-insoluble fractions extracted from transfected cells co-expressing HA-tagged human tau 3R1N and 4R1N with AD-tau seeds. Electron micrographs show fibrous structures positive for anti-HA antibody (*left*), pS396 antibody (*middle*) and TauC (*right*), that were labelled with the secondary antibody conjugated to 10 nm gold particles. Scale bar = 100 nm. (**C**) PHF-like tau filament positive for anti-HA antibody in sarkosyl-insoluble fractions extracted from transfected cells co-expressing HA-tagged human tau 3R1N and 4R1N with AD-tau seeds was labelled with secondary antibody conjugated to 10 nm gold particles. Scale bar = 50 nm. (**D**) Immunoelectron microscopy of sarkosyl-insoluble fractions extracted from transfected cells expressing FLAG-tagged human tau 3R1N (*left*), 4R1N (*middle*) and co-expressing FLAG-tagged human tau 3R1N and 4R1N (*right*) with AD-tau seeds. PHF-like tau structures positive for anti-FLAG antibody were labelled with secondary antibody conjugated to 10 nm gold particles. Scale bar = 50 nm.

We also examined the prion-like properties of tau aggregates amplified in SH-SY5Y cells with introduced patient-derived tau strains. To clarify the ultrastructural features of tau aggregates accumulated in SH-SY5Y cells, immunolabelling of the sarkosyl-insoluble fractions extracted from transfected cells using anti-HA, anti-FLAG, TauC and pS396 antibodies was performed. Abundant amyloid-like filamentous structures labelled with anti-HA, pS396 and TauC antibodies were observed in the insoluble fractions extracted from transfected cells expressing either HA-tagged 3R-FL or 4R-FL with introduced PiD-, PSP-, CBD- and AD-tau seeds (Fig. 6A and Supplementary Fig. 8A). HA-labelled filamentous structures with wide regions of 14.2 (±2.0, mean SD) nm, 13.0 (±1.2) nm, and 14.0 (±2.0) nm diameter and narrow regions of 6.7 (±1.2) nm, 4.6 (±0.8) nm and 5.9 (±0.6) nm diameter were observed in insoluble fractions from SH-SY5Y cells with introduced PiD-, PSP- and CBD-tau seeds, respectively (Fig. 6A and Supplementary Fig. 8A). Tau filaments extracted from cells with introduced PiD- and PSP-tau seeds resembled those extracted from the patients’ brains, but tau filaments from cells with introduced CBD-tau seeds were thinner than those in the brain. PHF-like structures were observed in transfected cells expressing either 3R-FL or 4R-FL with AD-tau seeds. These filaments showed wide regions of 17.9 (±2.6) nm and 19.0 (±1.8) nm diameter and narrow regions of 6.9 (±1.1) nm and 6.2 (±0.6) nm diameter, respectively, and were twisted with 94.9 (±9.8) nm and 93.8 (±10.0) nm periodicity (Fig. 6A and Supplementary Fig. 8A). PHF-like filaments positive for anti-HA, pS396 and TauC antibodies, which contained wide regions of 17.0 (±2.9) nm diameter and narrow regions of 7.0 (±1.3) nm diameter and were twisted with 83.7 (±3.7) nm periodicity, were also observed in the insoluble fraction extracted from transfected cells expressing both HA-tagged 3R-FL and 4R-FL (Fig. 6B and C). These HA-labelled tau filaments are akin to PHFs observed in the brain of patients with Alzheimer’s disease, suggesting that AD-tau seeds could work as a template to form PHF-like filaments in SH-SY5Y cells. Furthermore, strain-dependent seeded tau aggregation is induced not only in cells expressing HA-tagged tau, but also cells expressing FLAG-tagged tau. Introduction of AD-tau seeds into cells expressing FLAG-tagged 3R-FL or 4R-FL, or both resulted in the accumulation of phosphorylated insoluble FLAG-tagged tau (Supplementary Fig. 8B). Immunolabelling of these insoluble fractions revealed PHF-like filaments labelled with anti-FLAG antibody (Fig. 6D and Supplementary Fig. 9C). PHF-like filaments were seen in all cases of expression of FLAG-tagged tau, as observed in cells expressing HA-tagged tau. These fibrils showed wide regions of 20.6 (±2.1, mean SD) nm, 17.5 (±2.6) nm and 22.4 (±2.1) diameter and narrow regions of 6.4 (±0.7) nm, 6.3 (±1.8) nm and 7.9 (±1.2) nm diameter, respectively, and were twisted with 86.6 (±8.3) nm, 89.9 (±5.0) nm and 88.2 (±8.9) nm periodicity. Similarly, PiD-, PSP-, and CBD-tau seeds were added to cells expressing either FLAG-tagged 3R-FL or 4R-FL, and isoform-dependent seeded tau aggregation was detected, as observed in cells expressing HA-tagged tau (Supplementary Fig. 9A). No amyloid-like fibrous structure was observed in the insoluble fraction extracted from cells with introduced Huntington’s disease seeds (Supplementary Fig. 9B). FLAG-labelled amyloid-like filaments with wide regions of 14.7 (±1.9) nm, 13.8 (±1.3) nm and 14.1 (±1.1) nm diameter and narrow regions of 6.5 (±0.7) nm, 5.4 (±0.5) nm and 6.5 (±0.5) nm diameter were observed in cells with introduced PiD-, PSP- and CBD-tau seeds, respectively (Supplementary Fig. 9D). These results indicate that patient-derived tau seeds act as a template, and intracellular HA- or FLAG-tagged tau forms amyloid-like filaments similar to those derived from patients’ brains, which accumulate in the cells.

### Absence of phosphorylation at Ser262 of abnormal tau amplified in transfected cells with PiD-tau seeds

**Figure 7:**
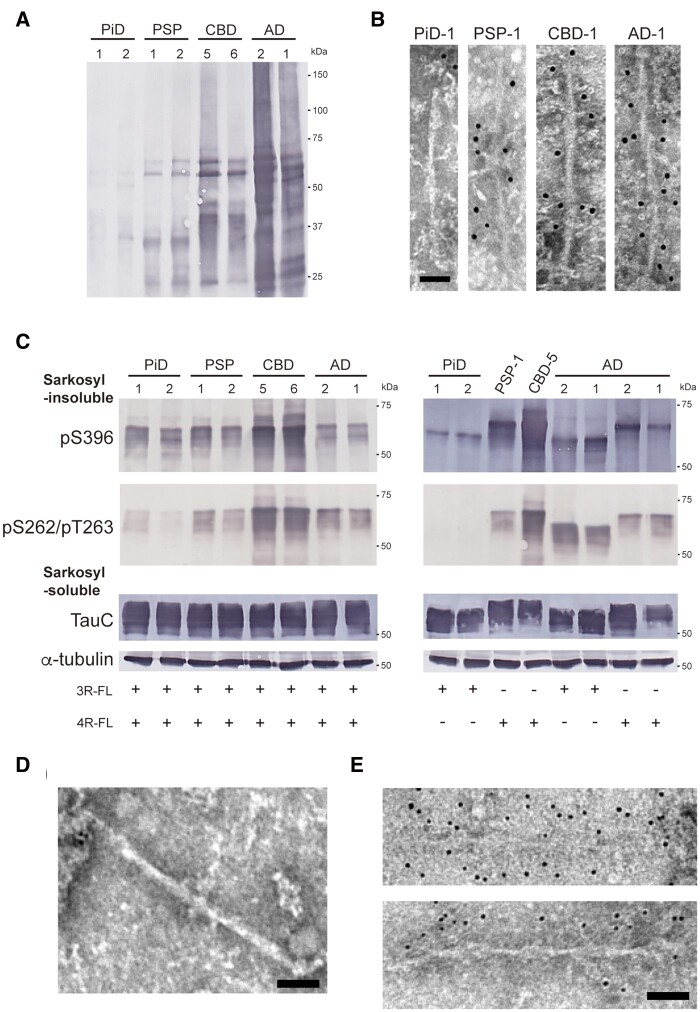
**Phosphorylation at Ser262 in insoluble tau extracted from SH-SY5Y cells transfected with patient-derived tau strains.** (**A**) Immunoblot analysis with pS262/pT263 antibody of sarkosyl-insoluble fractions prepared from brains of tauopathy patients. Insoluble tau derived from PSP, CBD, and Alzheimer’s disease cases shows pS262-positive bands, while insoluble tau derived from PiD cases is pS262-negative. Full-length blots are presented in the Supplementary material. (**B**) Immunoelectron microscopy of sarkosyl-insoluble fractions extracted from PiD, PSP, CBD and Alzheimer’s disease patients’ brains. Electron micrographs show fibrous structures derived from PiD negative for pSer262/pT263 antibody and fibrous structures derived from PSP, CBD and Alzheimer’s disease positive for pSer262/pT263 antibody, labelled with secondary antibody conjugated to 10 nm gold particles. Scale bar = 50 nm. (**C**) Sarkosyl-insoluble fractions extracted from patients’ brains (1 μl) were introduced into SH-SY5Y cells transiently co-expressing HA-tagged human tau 3R1N and 4R1N (*left*), and expressing HA-tagged human tau 3R1N or 4R1N (*right*). Immunoblot analysis of sarkosyl-insoluble fractions and sarkosyl-soluble fractions extracted from mock-transfected cells, and cells with introduced sarkosyl-insoluble fractions from two PiD cases, two PSP cases, two CBD cases and two Alzheimer’s disease cases. Insoluble tau was detected with pS396 and pS262/pT263 antibodies. Total tau was detected with TauC antibody. Full-length blots are presented in the Supplementary material. (**D**) Immunoelectron microscopy of sarkosyl-insoluble fractions extracted from transfected cells with PiD-tau seeds. Fibrous structure negative for pS262/pT263 antibody is shown. Scale bar = 50 nm. (**E**) Immunoelectron microscopy of sarkosyl-insoluble fractions extracted from transfected cells with AD-tau seeds. PHF-like tau structures positive for pS262/pT263 antibody, labelled with secondary antibody conjugated to 10 nm gold particles. Scale bar = 50 nm.

The tau aggregates derived from the brains of tauopathy patients contain diverse post-translational modifications. It has been reported that Ser262 is not phosphorylated in abnormal tau accumulated in the brain of PiD patients, probably because it is located in the core structure of tau filaments, where it is inaccessible.^41^^,^^42^ We performed immunoblotting and immunolabelling with pS262/pT263 antibody of patient-derived tau seeds used in this study, and confirmed that the PiD-tau seeds are pS262-negative (Fig. 7A andB). To examine whether insoluble tau extracted from cells with introduced PiD-tau seeds inherits pS262-negativity, immunoblotting and immunolabelling were performed using the insoluble fractions extracted from cells with introduced patient-derived tau strains. Immunoblotting of insoluble fractions extracted from transfected cells showed that insoluble tau from cells treated with PSP-, CBD-, and AD-tau seeds was pS262-positive, while that from cells treated with PiD-tau seeds was pS262-negative (Fig. 7C and Supplementary Fig. 7C). Furthermore, PHF-like filaments derived from cells with introduced AD-tau seeds were labelled with pS262/pT263 antibody, while filament structures derived from cells with introduced PiD-tau seeds were not (Fig. 7D and E). These results suggested that the abnormal tau amplified and accumulated in the transfected cells inherited the biochemical properties of the patient-derived tau strains.

### Inheritance of prion-like seeding properties through multiple passages of insoluble tau in SH-SY5Y cells

**Figure 8:**
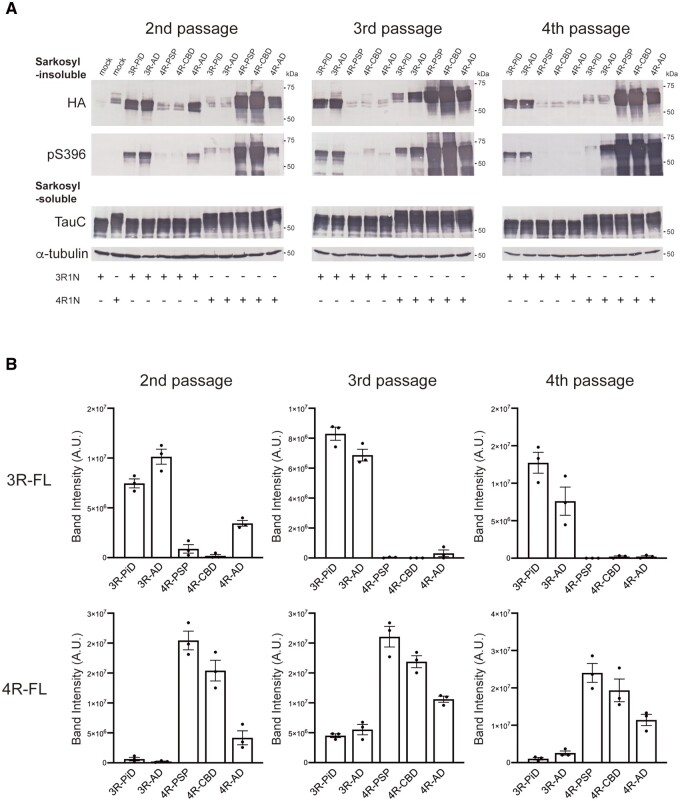
**Serial passages of insoluble tau extracted from transfected cells.** (**A**) Sarkosyl-insoluble fractions extracted from cells transfected with patient-derived tau strains were introduced into SH-SY5Y cells transiently expressing HA-tagged human tau 3R1N or 4R1N (second passage). The insoluble tau obtained after the second passage was further passed into SH-SY5Y cells twice more (third passage and fourth passage). Immunoblot analyses of sarkosyl-insoluble fractions and sarkosyl-soluble fractions extracted from cells transfected with 3R-PiD, 3R-AD, 4R-PSP, 4R-CBD and 4R-AD are shown. Insoluble tau was detected with anti-HA and pS396 antibodies. Total tau was detected with TauC antibody. Full-length blots are presented in the Supplementary material. (**B**) The band intensities of the anti-HA antibody immunoblots shown in **A** were quantified. The results are expressed as means ± SEM (*n* = 3).

Lastly, we investigated whether abnormal tau amplified in cells introduced with patient-derived tau seeds possesses seeding activity and inherits the prion-like properties of the original seeds. The insoluble fraction extracted from the transfected cells was introduced as secondary seeds into SH-SY5Y cells expressing HA-tagged 3R-FL or 4R-FL. Secondary seeds derived from cells introduced with patient-derived tau seeds caused the strain-dependent accumulation of insoluble tau in the same manner as the original seeds (Fig. 8). Secondary PiD-tau (3R-PiD) and AD-tau (3R-AD) seeds derived from cells expressing 3R-FL induced seeded aggregation of 3R tau. Secondary PSP-tau (4R-PSP), CBD-tau (4R-CBD) and AD-tau (4R-AD) seeds derived from cells expressing 4R-FL involved only 4R tau in seeded aggregation. Intriguingly, secondary 3R-PiD seeds extracted from cells with introduced unpurified PiD-2 seeds induced seeded aggregation of only 3R tau, and accumulation of 4R tau was not detected (Fig. 8). This supports the idea that accumulation of insoluble 4R tau induced by tau seeds derived from the PiD-2 case shown in Figs 2–4 was due to contamination. In addition, the strain-specific tau aggregation induced by cell-derived insoluble tau was maintained after two additional passages (Fig. 8). These results indicate that the tau seeds amplified in the cells inherit the prion-like seeding activity of the original seeds, and the isoform specificity is also retained in subsequent seeding.

## Discussion

We investigated seeded tau aggregation caused by tau strains derived from the brains of patients with tauopathy and the prion-like properties of patient-derived tau strains in cellular model. Pathogenic tau seeds induced disease-specific seeded tau aggregation and filament formation in SH-SY5Y cells. The 3R and 4R tau isoforms, which form the frameworks of tau filaments accumulated in the brains of patients with tauopathies, are key determinants of the neuropathology. PiD-, PSP- and CBD-tau seeds used in this study induced isoform-dependent tau aggregation in SH-SY5Y cells expressing 3R-FL or 4R-FL individually, or co-expressing both 3R-FL and 4R-FL (Figs 2A, B and 4). This isoform-dependent tau aggregation is also reproduced in SH-SY5Y cells with introduced synthetic 3R1N and 4R1N fibrils.^43^ On the other hand, AD-tau seeds composed of six tau isoforms recruited both 3R tau and 4R tau into abnormal tau, in contrast to a previous report that AD-tau seeds were unable to infect HEK293 cells expressing either 3R tau or 4R tau (Figs 2A, B, 4 and 5C–F).^44^ These results indicate that the pathogenic tau seeds extracted from patients’ brains retain structural information as a template for tau substrates. Furthermore, template-dependent tau filament formation was observed in SH-SY5Y cells with introduced patient-derived tau strains (Fig. 6 and Supplementary Figs 8 and 9). Interestingly, AD-tau seeds showed different recruitment efficiencies for 3R-FL and 4R-FL substrates (Fig. 5C and D). These differences in seeding activity may be due to the weak interaction of 3R tau with microtubules compared to that of 4R tau, which affects the rate and amount of intracellular accumulation of insoluble tau (Fig. 2A and B).^45^ However, high seeding activity of AD-tau seeds towards 3R tau has also been reported in an *in vitro* assay using real-time quaking-induced conversion (RT-QuIC).^46^ This assay is an experimental model that is not affected by binding to microtubules or other cellular environments, and the high recruitment efficiency of AD-tau seeds for 3R tau substrate in our cellular model indicates that the difference in conformation between normal 3R tau and 4R tau alters the interaction with AD-tau seeds.

What drives the isoform specificity of pathogenic tau seeds derived from human tauopathies? Atomic-level structural studies of tau filaments extracted from human tauopathies strongly support the strain-dependent nature of seeded tau aggregation observed in our cellular model. Cryo-EM analysis revealed that tau filaments extracted from brains of Alzheimer’s disease, chronic traumatic encephalopathy, PiD and CBD patients had disease-specific core structures, and patients in the same disease group showed identical folds.^42^^,^^47–50^ The core region of Alzheimer’s disease filaments is G273-E380 in 3R tau and G304-E380 in 4R tau, which covers the microtubule-binding domain including V306-K311, that is known to be important for tau aggregation.^51^ The structural evidence that the Alzheimer’s disease fold involves the common parts of 3R tau and 4R tau may explain why PHF-like filaments induced by AD-tau seeds were observed not only in cells expressing both 3R tau and 4R tau, but also in cells expressing 3R tau or 4R tau individually (Fig. 6C, D and Supplementary Fig. 9C). *In vitro* PHF-like filament formation of truncated tau C291-P397 in the absence of heparin also supports the idea that PHF-like filament formation induced by AD-tau seeds is not restricted by the substrate tau isoforms.^52^^,^^53^ The PiD and CBD folds have the core regions of K254-F378 containing R1, and K274-E380 containing the last single amino acid of R1 and the whole region of R2, respectively, and both cover the core region of the Alzheimer’s disease fold consisting of R3 and R4. These folds can account for the isoform specificity of patient-derived tau strains, i.e. that AD-tau seeds recruit both 3R tau and 4R tau for seeded aggregation, while PiD- and CBD-tau seeds selectively recruit 3R tau or 4R tau. It is important for the isoform specificity that the core structures of the PiD and CBD folds contain K254-G272 and K274-G303, respectively. This is supported by the ability of trypsinized patient-derived tau seeds to induce strain-specific tau aggregation (Fig. 3). Template-substrate mismatches caused by a lack or an excess of microtubule-binding repeats in substrates would lead to structural instability and would not promote polymerization. A substrate that covers the entire region of the template is essential for prion-like templated amplification, and partial identity of the amino acid sequence is insufficient. Although the core region of the PSP fold has not yet been determined, most of the core regions identified by cryo-EM analysis correspond to the trypsin-resistant regions of sarkosyl-insoluble tau derived from the brains of patients with tauopathy. Different trypsin-resistant core regions have been identified between CBD and PSP, indicating that the conformational difference between PSP- and CBD-tau seeds causes the observed distinct seeding activity.^25^

Pathogenic tau seeds derived from human tauopathies showed disease-specific seeding activities towards full-length tau substrates (Fig. 2A and B). In our cellular model, PiD-tau seeds exhibited lower activity than AD-tau seeds towards the 3R-FL tau substrate. On the other hand, the seeding activity for the 4R-FL tau substrate was in the order of CBD-tau seeds > PSP-tau seeds > AD-tau seeds (Fig. 5). As previously mentioned, it is clear that the disease-specific core structures of tau filaments affect the prion-like seeding activity, but what factors cause the formation of distinct tau strains with different ordered structures? One of these factors is the difference in the cellular environment in which normal tau forms the primary seeds. CBD- and PSP-tau seeds, which are characterized by accumulation of abnormal tau in glial cells, showed higher seeding activity than AD-tau seeds that accumulate in neurons (Fig. 5). Furthermore, the higher seeding activity of CBD-tau seeds compared to PSP seeds suggests that the formation of distinct 4R tau strains may depend on whether 4R tau aggregation occurs in the distal dendrite or central parts of astrocytes (Fig. 5A and B).^10^ It has been reported that tau seeds derived from the brain of a globular glial tauopathy case in which 4R tau accumulated in glial cells exhibited higher seeding activity than tau seeds derived from other tauopathies.^54^ High prion-like seeding activity of abnormal α-syn derived from multiple system atrophy cases characterized by accumulation of α-syn in glial cells has also been reported, indicating that abnormal proteins derived from glial cells tend to show higher seeding activity than those derived from neurons.^37^^,^^55^^,^^56^ Cryo-EM analysis of patient-derived filaments revealed the presence of cofactors besides tau and α-syn in the core structures of filaments derived from chronic traumatic encephalopathy, CBD and multiple system atrophy cases, but not Alzheimer’s disease and PiD cases.^49^^,^^50^^,^^57^ Although it is not yet clear exactly what is the key difference in the cellular environment between neurons and glial cells, the cofactors incorporated into these core structures are derived from glial cells and may contribute to the formation of distinct strains. Regarding the CTFs of abnormal tau linked to the structural diversity of patient-derived tau filaments, insoluble tau derived from cells introduced with patient-derived tau strains showed banding patterns of CTFs similar to those of the patient-derived CTFs, though the banding patterns of CTFs derived from cells with introduced PSP- and CBD-tau seeds were indistinguishable (Fig. 2C and Supplementary Fig. 2D). The reason for this may be that the SH-SY5Y cells used in this study are derived from neurons, and this is consistent with the idea that certain differences in the cellular environment influence the conformation of tau filaments formed in SH-SY5Y cells. It is necessary to investigate whether these disease-specific CTFs are reproduced in glial cells and mouse brain. The effect of cellular environment on template-dependent tau filament formation could also be clarified by cryo-EM analysis of tau filaments derived from cells introduced with patient-derived tau strains and comparison of their structures with those of patient-derived tau filaments. Furthermore, post-translational modifications may be involved in the formation of distinct tau strains, because abnormal tau species derived from human tauopathies show different post-translational modification patterns depending on the disease.^58^ We confirmed that pS262-negativity in PiD cases is inherited in cellular seeded aggregation (Fig. 7). Moreover, the predominance of tau isoforms in neurons and glial cells may be important for the formation of tau strains that selectively recruit normal 3R tau or 4R tau for conversion to abnormal forms. The formation of the PiD fold may be attributed to the predominant expression of 3R tau in interneurons, in where Pick bodies are frequently observed.^59^ Similarly, the accumulation of 4R tau in PSP and CBD cases may be related to the predominant expression of 4R tau in glial cells. In addition, we performed serial passage experiments to investigate whether various strains formed in the patient’s brain change as they are amplified and spread in the brain. The tau seeds extracted from transfected cells with patient-derived tau seeds or cell-derived tau seeds retained similar isoform specificity to the original seeds (Fig. 8). It is suggested that the prion-like seeding properties of the original seeds are maintained during the intracerebral expansion of the pathology.

Thus, we have shown that pathogenic tau seeds from human tauopathies cause strain-dependent seeded tau aggregation in SH-SY5Y cells. We wish to emphasize the importance of using patient-derived pathogenic seeds in prion-like propagation experimental models. The method for preparing tau filaments from the patient’s brain is also important, and the use of highly purified patient-derived protein seeds greatly contributes to the accurate reproduction of the patient’s brain pathology, as shown by the effect of contaminants in PiD-2 seeds on seeded aggregation (Supplementary Fig. 3A–D). Furthermore, we clarified that the difference in prion-like seeding properties of tau from human tauopathy was observed in cells expressing full-length tau (Fig. 5). The inadequacy of CTFs containing only the microtubule-binding domain is supported by studies showing that the core structures of patient-derived tau filaments are composed of microtubule-binding domain and the additional C-terminal region. It has also been reported that the FRET-based tau seeding assay does not replicate template-dependent tau filament formation.^60^ Therefore, our cellular model of seeded tau aggregation using biochemically and ultrastructurally well-characterized pathogenic tau seeds derived from human tauopathies is expected to be useful as a prion-like propagation model that mimics the pathogenesis of sporadic tauopathy.

## Supplementary Material

awab091_Supplementary_Data

## Abbreviations

CBD: corticobasal degeneration
CTF: C-terminal fragment
PHF: paired helical filament
PiD: Pick’s disease
PSP: progressive supranuclear palsy
