# Supplementary material for: Human tauopathy-derived tau strains determine the substrates recruited for templated amplification
Source: Brain. 2021 Mar 9;144(8):2333–48. doi: 10.1093/brain/awab091 (PMC8418341; doi:10.1093/brain/awab091)
Supplement: awab091_Supplementary_Data [file awab091_supplementary_data.zip › awab091-suppl_data/brain-2020-01854-File010.pdf]

A

Diagnosis	Case number	Gender	Age at death	Duration (year)	Brain (g)	PMI (hr)
HD	1	M	33	6	1440	N/A
HD	2	M	45	N/A	1150	N/A
PiD	1	M	56	10	1150	N/A
PiD	2	F	62	10	928	N/A
PiD	3	M	71	N/A	1000	8
PiD	4	N/A	N/A	N/A	N/A	N/A
PSP	1	F	88	12	N/A	24
PSP	2	M	78	18	N/A	19
PSP	3	M	82	6	1140	43
PSP	4	M	82	N/A	1280	3
CBD	1	M	83	N/A	1096	N/A
CBD	2	F	74	6	899	N/A
CBD	3	M	65	N/A	1260	12
CBD	4	M	51	N/A	1340	6
CBD	5	M	73	N/A	1200	3
CBD	6	F	74	35	N/A	N/A
AD	1	M	56	4	1358	N/A
AD	2	F	65	9	1165	N/A
AD	3	N/A	N/A	N/A	N/A	N/A
AD	4	F	94	15	983	11
AD	5	M	85	N/A	1146	8

B

Case number	Brain region	Total tau (ng/mL)
HD-1	frontal lobe	1085.20 (± 9.86)
HD-2	frontal lobe	1004.28 (± 60.00)
PiD-1	frontal lobe	3366.27 (± 416.80)
PiD-2	frontal lobe	1768.66 (± 16.86)
PiD-3	occipital lobe	3021.70 (± 192.74)
PiD-4	frontal lobe	379.40 (± 45.84)
PSP-1	frontal lobe	3822.74 (± 501.26)
PSP-2	frontal lobe	5835.75 (± 293.63)
PSP-3	frontal lobe	4393.95 (± 263.49)
PSP-4	frontal lobe	2204.49 (± 169.14)
CBD-1	frontal lobe	3426.46 (± 72.13)
CBD-2	frontal lobe	13873.72 (± 990.06)
CBD-3	frontal lobe	993.21 (± 105.14)
CBD-4	frontal lobe	516.29 (± 59.31)
CBD-5	frontal lobe	11542.00 (± 665.14)
CBD-6	putamen	14576.14 (± 400.86)
AD-1	temporal lobe	24007.57 (± 671.95)
AD-2	temporal lobe	41352.39 (± 1943.97)
AD-3	temporal lobe	64668.77 (± 21.55)
AD-4	frontal lobe	2870.65 (± 15.76)
AD-5	frontal lobe	3083.02 (± 4.49)

Supplemental Table. Post-mortem brain tissues used in this study

A. Neuropathological information for post-mortem brain tissues used in this study

B. Total tau concentrations in sarkosyl-insoluble fractions extracted from patients' brains

HD; Huntinton's disease, PiD; Pick's disease, PSP; progressive supranuclear palsy, CBD;corticobasal degeneration, AD; Alzheimer's disease

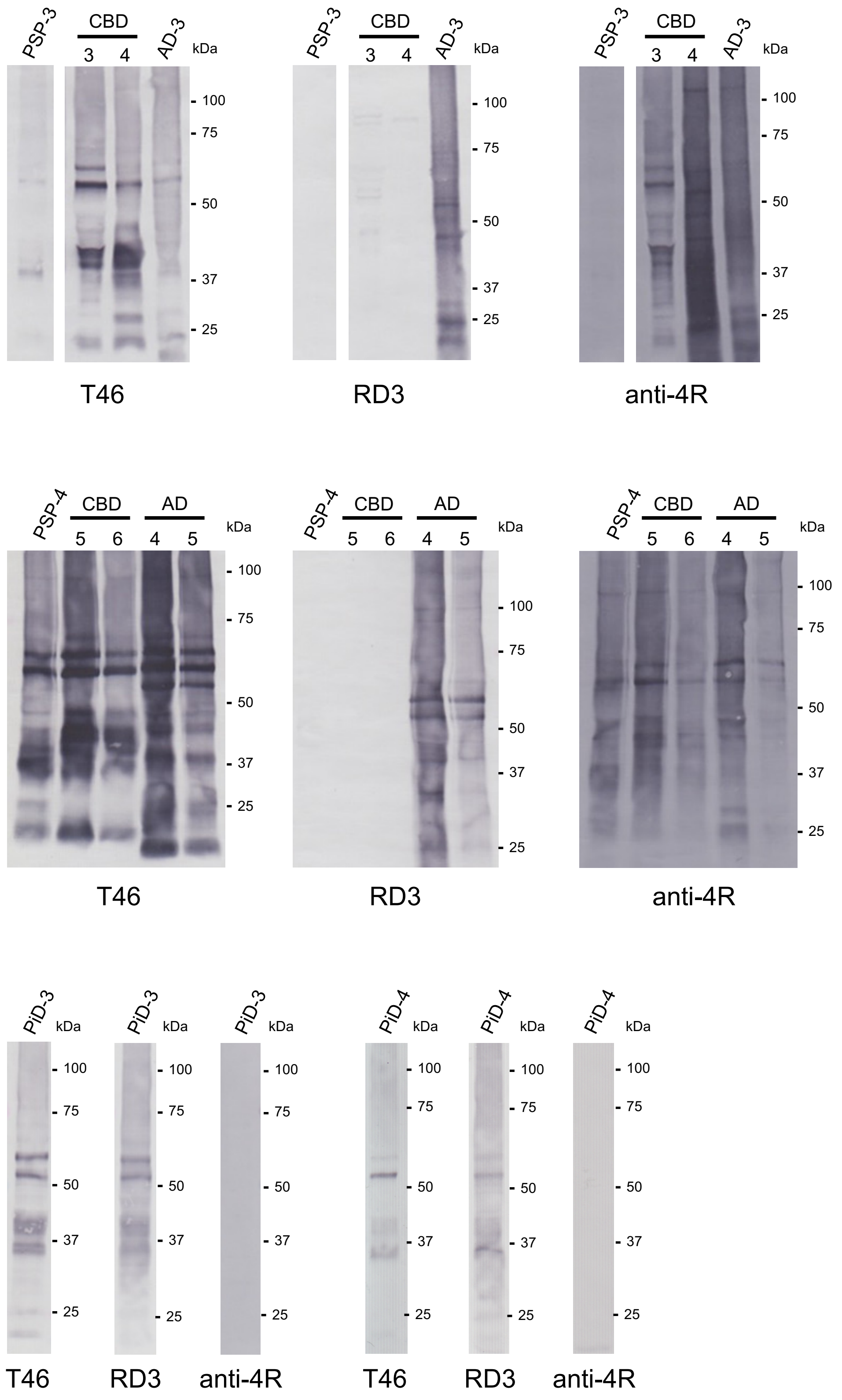

Supplemental Fig 1. Biochemical characterization of abnormal tau extracted from brains of patients with tauopathies

Sarkosyl-insoluble fractions prepared from patients' brains used in this study were analyzed by immunoblotting with T46, RD3 and anti-4R antibodies. Full-length blots are presented in Supplementary material.

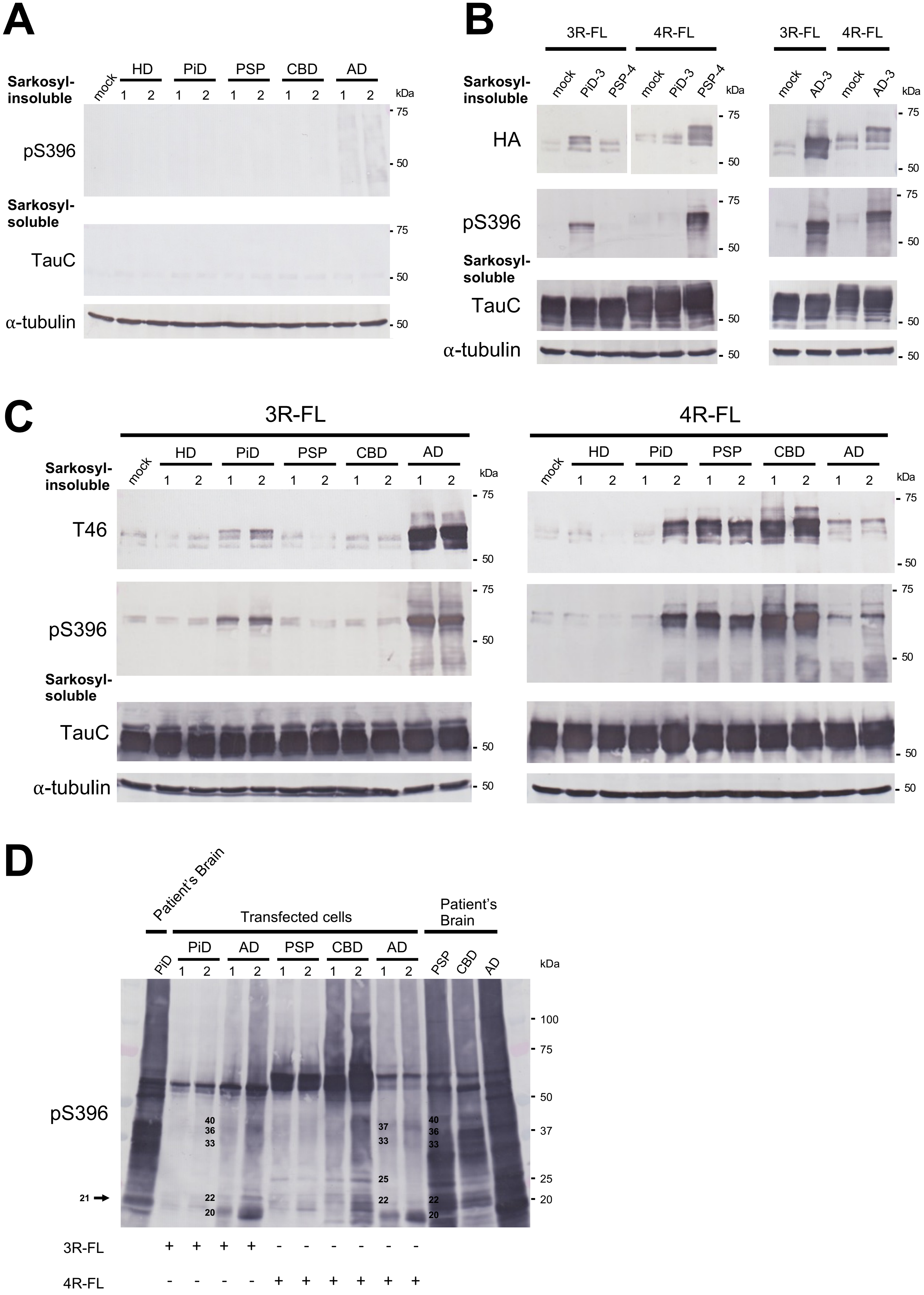
A
Sarkosyl-insoluble
mock
HD
PiD
PSP
CBD
AD
1 2
kDa
pS396
Sarkosyl-soluble
TauC
α-tubulin
75
50
B
3R-FL
4R-FL
mock
PiD-3
PSP-4
AD-3
HA
pS396
C
3R-FL
4R-FL
T46
D
Patient's Brain
Transfected cells
Patient's Brain
100
37
25
20
40
36
33
22
21
3R-FL
4R-FL
+
-

Supplemental Fig 2. Introduction of patient-derived tau seeds into SH-SY5Y cells expressing full-length tau

A, Sarkosyl-insoluble fractions extracted from patients' brains (1 μl) were introduced into SH-SY5Y cells without transient expression of tau. Immunoblot analysis of sarkosyl-insoluble fractions and sarkosyl-soluble fractions extracted from mock-transfected cells, and cells with introduced sarkosyl-insoluble fractions from 2 PiD cases, 2 PSP cases, 2 CBD cases and 2 AD cases. Insoluble tau was detected with pS396 antibody. Total tau was detected with TauC antibody. Full-length blots are presented in Supplementary material.

B, Sarkosyl-insoluble fractions extracted from PiD-3, PSP-4 and AD-3 cases (1 μl) were introduced into SH-SY5Y cells transiently expressing HA-tagged human tau 3R1N or 4R1N. Immunoblot analysis of sarkosyl-insoluble fractions and sarkosyl-soluble fractions extracted from mock-transfected cells, and cells treated with sarkosyl-insoluble fractions from PiD-3, PSP-4 and AD-3 cases. Insoluble tau was detected with anti-HA and pS396 antibodies. Total tau was detected with TauC antibody. Full-length blots are presented in Supplementary material.

C, Sarkosyl-insoluble fractions extracted from patients' brains (1 μl) were introduced into SH-SY5Y cells transiently expressing non-tagged human tau 3R1N or 4R1N. Immunoblot analysis of sarkosyl-insoluble fractions and sarkosyl-soluble fractions extracted from mock-transfected cells, and cells with introduced sarkosyl-insoluble fractions from 2 HD cases, 2 PiD cases, 2 PSP cases, 2 CBD cases and 2 AD cases. Insoluble tau was detected with T46 and pS396 antibodies. Total tau was detected with TauC antibody. Full-length blots are presented in Supplementary material.

D, Immunoblot analysis of sarkosyl-insoluble fractions prepared from transfected cells expressing non-tagged tau and tau from patients' brains. C-Terminal tau fragments were detected by pS396 antibody. Full-length blots are presented in Supplementary material.

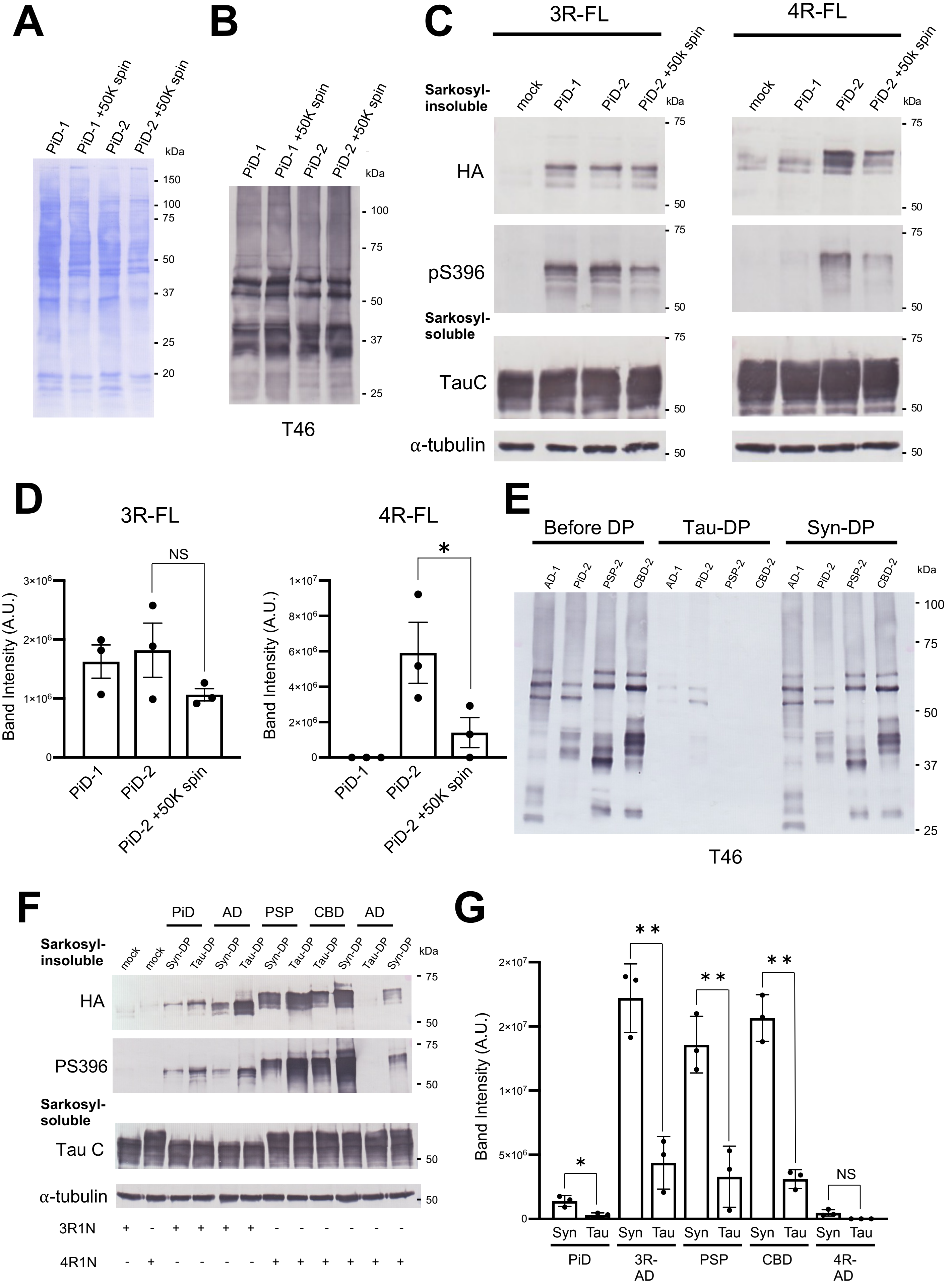
A
PiD-1
PiD-1 +50K spin
PiD-2
PiD-2 +50K spin
kDa
150
100
75
50
37
25
20
B
PiD-1
PiD-1 +50K spin
PiD-2
PiD-2 +50K spin
kDa
100
75
50
37
25
T46
C
3R-FL
4R-FL
Sarkosyl-insoluble
mock
PiD-1
PiD-2
PiD-2 +50k spin
kDa
HA
pS396
Sarkosyl-soluble
TauC
α-tubulin
75
50
D
3R-FL
4R-FL
NS
*
Band Intensity (A.U.)
3×10^6
2×10^6
1×10^6
0
1×10^7
8×10^6
6×10^6
4×10^6
2×10^6
PiD-1
PiD-2
PiD-2 +50K spin
E
Before DP
Tau-DP
Syn-DP
AD-1
PiD-2
PSP-2
CBD-2
kDa
100
75
50
37
25
T46
F
PiD
AD
PSP
CBD
AD
Sarkosyl-insoluble
mock
mock
Syn-DP
Tau-DP
kDa
HA
PS396
Sarkosyl-soluble
Tau C
α-tubulin
75
50
3R1N
4R1N
+
-
G
Band Intensity (A.U.)
2×10^7
1×10^7
5×10^6
0
* *
*
NS
Syn
Tau
PiD
3R-AD
PSP
CBD
4R-AD

Supplemental Fig 3. The effect of contaminants in sarkosyl-insoluble fractions derived from patients' brains on seeded aggregation

A, CBB staining of sarkosyl-insoluble fractions derived from 2 PiD cases before and after additional ultracentrifugation. Full-length blots are presented in Supplementary material.

B, Immunoblot analyses of PiD-tau seeds used in the seeding experiments shown in C and D. Sarkosyl-insoluble full-length tau (60 and 64 kDa) and C-terminal tau fragments were detected with T46 antibody. Full-length blots are presented in Supplementary material.

C, Sarkosyl-insoluble fractions from 2 PiD cases (total tau: 2 ng) before and after additional ultracentrifugation were introduced into SH-SY5Y cells transiently expressing HA-tagged human tau 3R1N (left) or 4R1N (right). Immunoblot analyses of sarkosyl-insoluble fractions and sarkosyl-soluble fractions extracted from mock-transfected cells, and cells with introduced PiD-tau seeds. Insoluble tau was detected with anti-HA and pS396 antibodies. Total tau was detected with TauC antibody. Full-length blots are presented in Supplementary material.

D, Quantification of the band intensities of the immunoblots with anti-HA antibody shown in C. The results are expressed as means ± SEM (n=3). *P < 0.05; Student's *t*-test against the value of PiD-2.

E, Immunoblot analysis of the sarkosyl-insoluble fractions before and after immunodepletion of tau (Tau-DP) or α-syn (Syn-DP). Full-length tau and C-terminal tau fragments were detected with T46 antibody. Full-length blots are presented in Supplementary material.

F, Tau- or α-syn-immunodepleted samples were introduced into SH-SY5Y cells transiently expressing HA-tagged human tau 3R1N or 4R1N. Immunoblot analyses of sarkosyl-insoluble fractions and sarkosyl-soluble fractions extracted from mock-transfected cells and cells with introduced immunodepleted samples. Insoluble tau was detected with anti-HA and pS396 antibodies. Total tau was detected with TauC antibody. Full-length blots are presented in Supplementary material.

G, Quantification of the band intensities of the immunoblots with anti-HA antibody shown in F. The results are expressed as means ± SEM (n=3). *P < 0.05; **P < 0.01; Student's *t*-test against the value of Syn-DP.

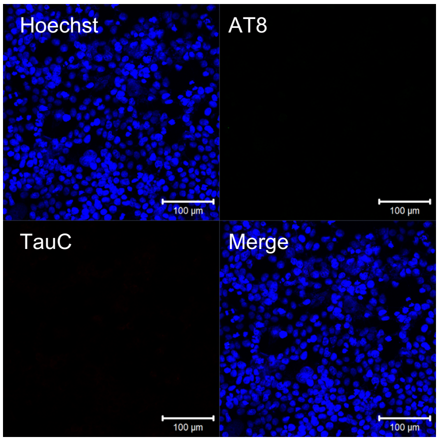

Blank

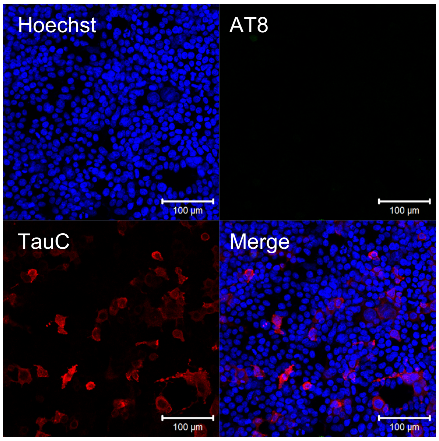

3R-FL + 4R-FL

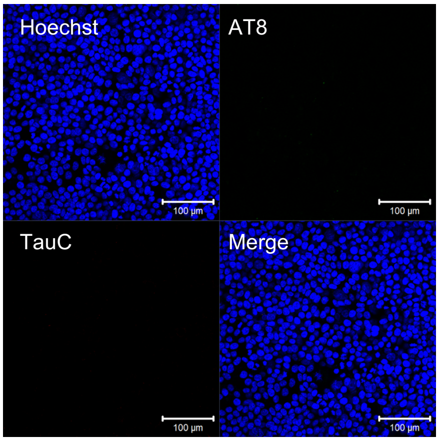

Blank + AD

Supplemental Fig 4. Immunohistochemistry of transfected SH-SY5Y cells without transient expression of tau

Mock-SH-SY5Y cells (Blank), SH-SY5Y cells transiently co-expressing human tau 3R1N and 4R1N (3R-FL + 4R-FL), and transfected SH-SY5Y cells without transient expression of tau with sarkosyl-insoluble fractions from an AD case (Blank + AD) were fixed and immunostained with AT8 (green) and TauC (red) antibodies. Scale bar, 100 µm.

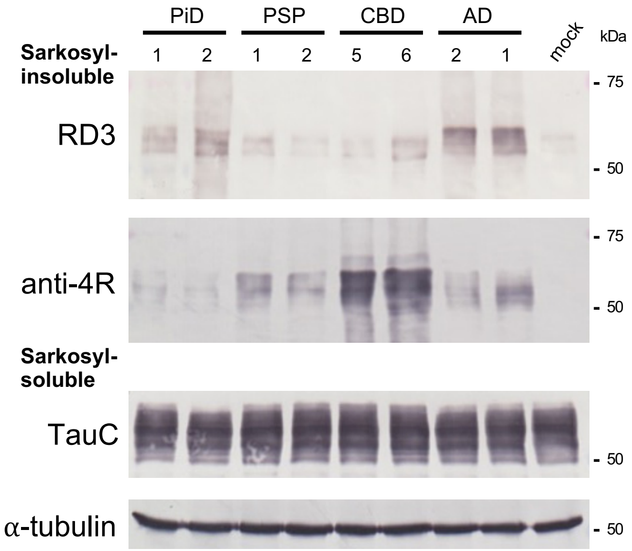

Supplemental Fig 5. Isoform-dependent seeded tau aggregation in SH-SY5Y cells co-expressing 3R and 4R tau

Sarkosyl-insoluble fractions extracted from patients' brains (1 μl) were introduced into SH-SY5Y cells transiently co-expressing HA-tagged human tau 3R1N and 4R1N. Immunoblot analysis of sarkosyl-insoluble fractions and sarkosyl-soluble fractions extracted from mock-transfected cells, and cells with introduced sarkosyl-insoluble fractions from 2 PiD cases, 2 PSP cases, 2 CBD cases and 2 AD cases. Insoluble 3R tau or 4R tau were detected with RD3 or anti-4R antibodies, respectively. Total tau was detected with TauC antibody. The results of quantification of the band intensities of immunoblots with RD3 and anti-4R antibodies are shown in Fig.4B. Full-length blots are presented in Supplementary material.

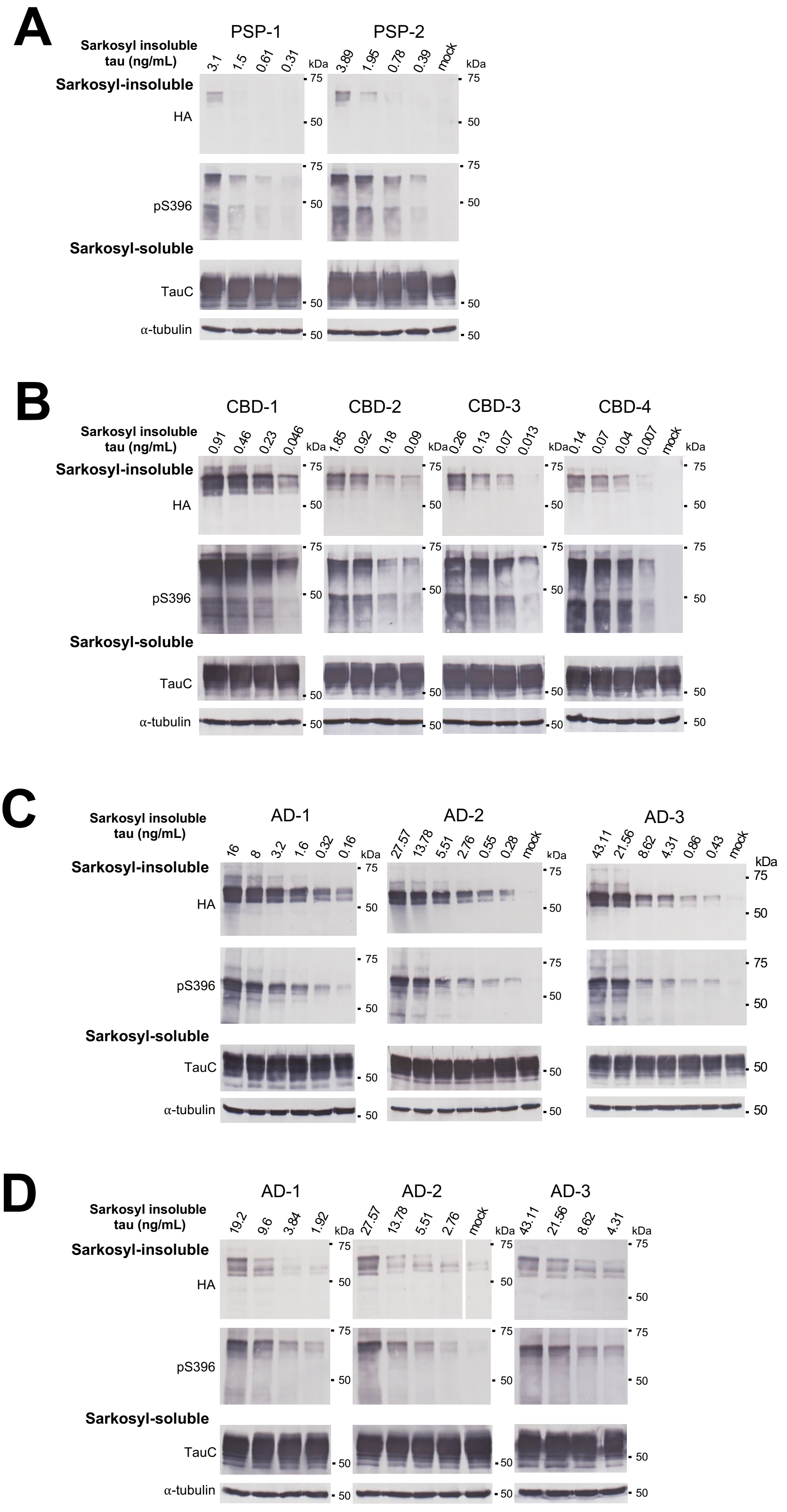
A
PSP-1
PSP-2
Sarkosyl insoluble tau (ng/mL)
3.1
1.5
0.61
0.31
3.89
1.95
0.78
0.39
mock
kDa
Sarkosyl-insoluble
HA
pS396
Sarkosyl-soluble
TauC
α-tubulin
75
50
B
CBD-1
CBD-2
CBD-3
CBD-4
Sarkosyl insoluble tau (ng/mL)
0.91
0.46
0.23
0.046
1.85
0.92
0.18
0.09
0.26
0.13
0.07
0.013
0.14
0.07
0.04
0.007
mock
kDa
Sarkosyl-insoluble
HA
pS396
Sarkosyl-soluble
TauC
α-tubulin
75
50
C
AD-1
AD-2
AD-3
Sarkosyl insoluble tau (ng/mL)
16
8
3.2
1.6
0.32
0.16
27.57
13.78
5.51
2.76
0.55
0.28
mock
43.11
21.56
8.62
4.31
0.86
0.43
mock
kDa
Sarkosyl-insoluble
HA
pS396
Sarkosyl-soluble
TauC
α-tubulin
75
50
D
AD-1
AD-2
AD-3
Sarkosyl insoluble tau (ng/mL)
19.2
9.6
3.84
1.92
27.57
13.78
5.51
2.76
mock
43.11
21.56
8.62
4.31
kDa
Sarkosyl-insoluble
HA
pS396
Sarkosyl-soluble
TauC
α-tubulin
75
50

Supplemental Fig 6. Prion-like seeding activities of serial dilutions of insoluble fractions of PSP, CBD and AD cases in SH-SY5Y cells

A, Sarkosyl-insoluble fractions extracted from 2 PSP cases were diluted and introduced into SH-SY5Y cells transiently expressing HA-tagged human tau 4R1N. Immunoblot analyses of sarkosyl-insoluble fractions and sarkosyl-soluble fractions extracted from cells transfected with serial dilutions of PSP-1 and PSP-2 are shown. Insoluble tau was detected with anti-HA and pS396 antibodies. Total tau was detected with TauC antibody. Full-length blots are presented in Supplementary material.

B, Sarkosyl-insoluble fractions extracted from 4 CBD cases were diluted and introduced into SH-SY5Y cells transiently expressing HA-tagged human tau 4R1N. Immunoblot analyses of sarkosyl-insoluble fractions and sarkosyl-soluble fractions extracted from transfected cells with serial dilutions of CBD 1-4 are shown. Insoluble tau was detected with anti-HA and pS396 antibodies. Total tau was detected with TauC antibody. Full-length blots are presented in Supplementary material.

C, Sarkosyl-insoluble fractions extracted from 3 AD cases were diluted and introduced into SH-SY5Y cells transiently expressing HA-tagged human tau 3R1N. Immunoblot analyses of sarkosyl-insoluble fractions and sarkosyl-soluble fractions extracted from transfected cells with serial dilutions of AD 1-3 are shown. Insoluble tau was detected with anti-HA and pS396 antibodies. Total tau was detected with TauC antibody. Full-length blots are presented in Supplementary material.

D, Sarkosyl-insoluble fractions extracted from 3 AD cases were diluted and introduced into SH-SY5Y cells transiently expressing HA-tagged human tau 4R1N. Immunoblot analyses of sarkosyl-insoluble fractions and sarkosyl-soluble fractions extracted from transfected cells with serial dilutions of AD 1-3 are shown. Insoluble tau was detected with anti-HA and pS396 antibodies. Total tau was detected with TauC antibody. Full-length blots are presented in Supplementary material.

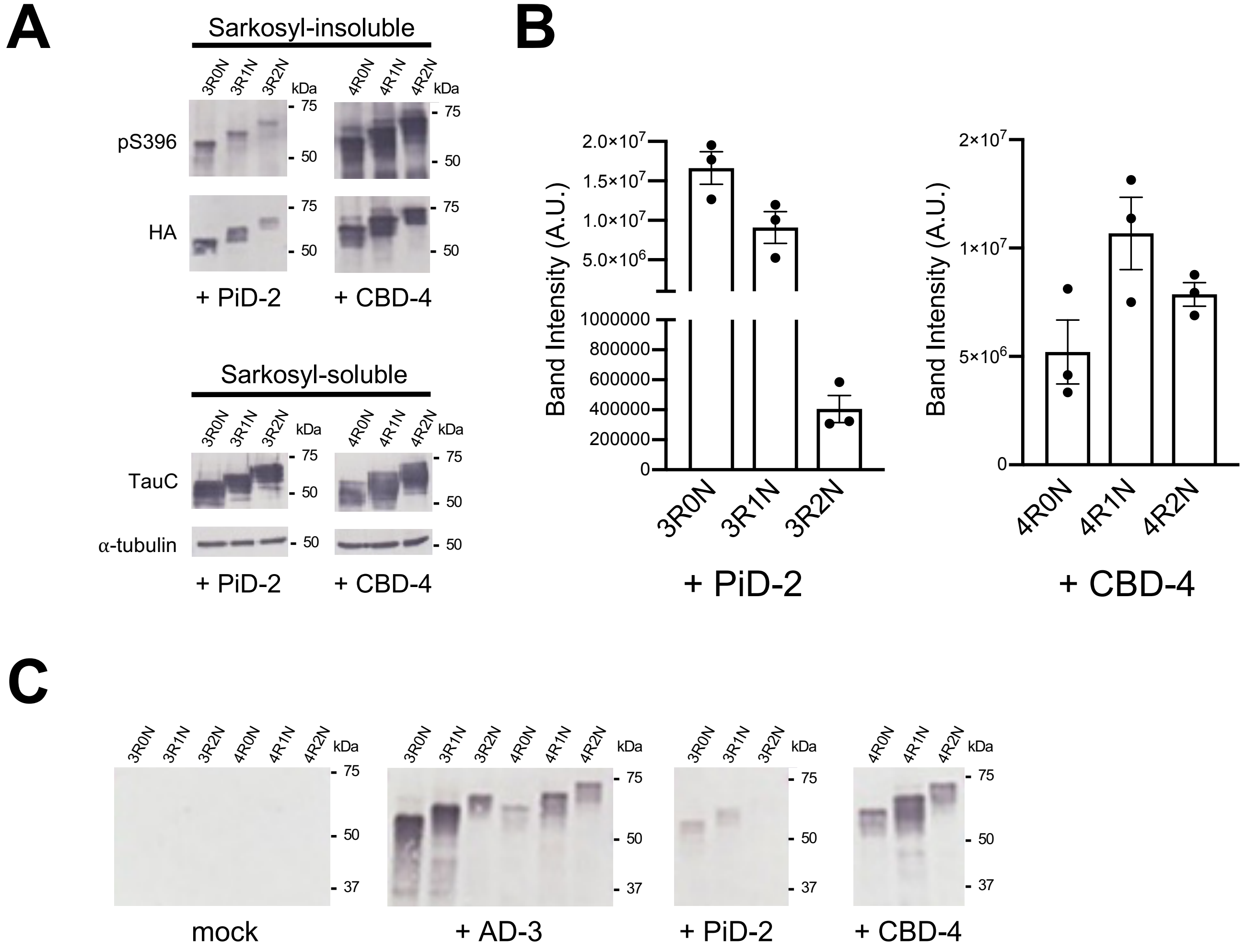

Supplemental Fig 7. Seeded tau aggregation in SH-SY5Y cells expressing various tau isoforms

A, Sarkosyl-insoluble fractions prepared from PiD-2 and CBD-4 cases (1 µl) were introduced into SH-SY5Y cells transiently expressing HA-tagged human tau 3R0N, 3R1N and 3R2N or 4R0N, 4R1N and 4R2N, respectively. Immunoblot analysis of sarkosyl-insoluble fractions and sarkosyl-soluble fractions extracted from transfected cells with sarkosyl-insoluble fractions from PiD-2 and CBD-4 cases. Insoluble tau was detected with pS396 and anti-HA antibodies. Total tau was detected with TauC antibody. Full-length blots are presented in Supplementary material.

B, The band intensities of the immunoblots with anti-HA antibody shown in A were quantified. The results are expressed as means ± SEM (n=3).

C, Immunoblot analysis of sarkosyl-insoluble fractions extracted from cells transfected with sarkosyl-insoluble fractions from AD-3, PiD-2 and CBD-4 cases shown in Fig. 5E and Fig. S7A. Immunoreactivites for pS262 of insoluble tau were detected with pS262/pT263 antibody. Full-length blots are presented in Supplementary material.

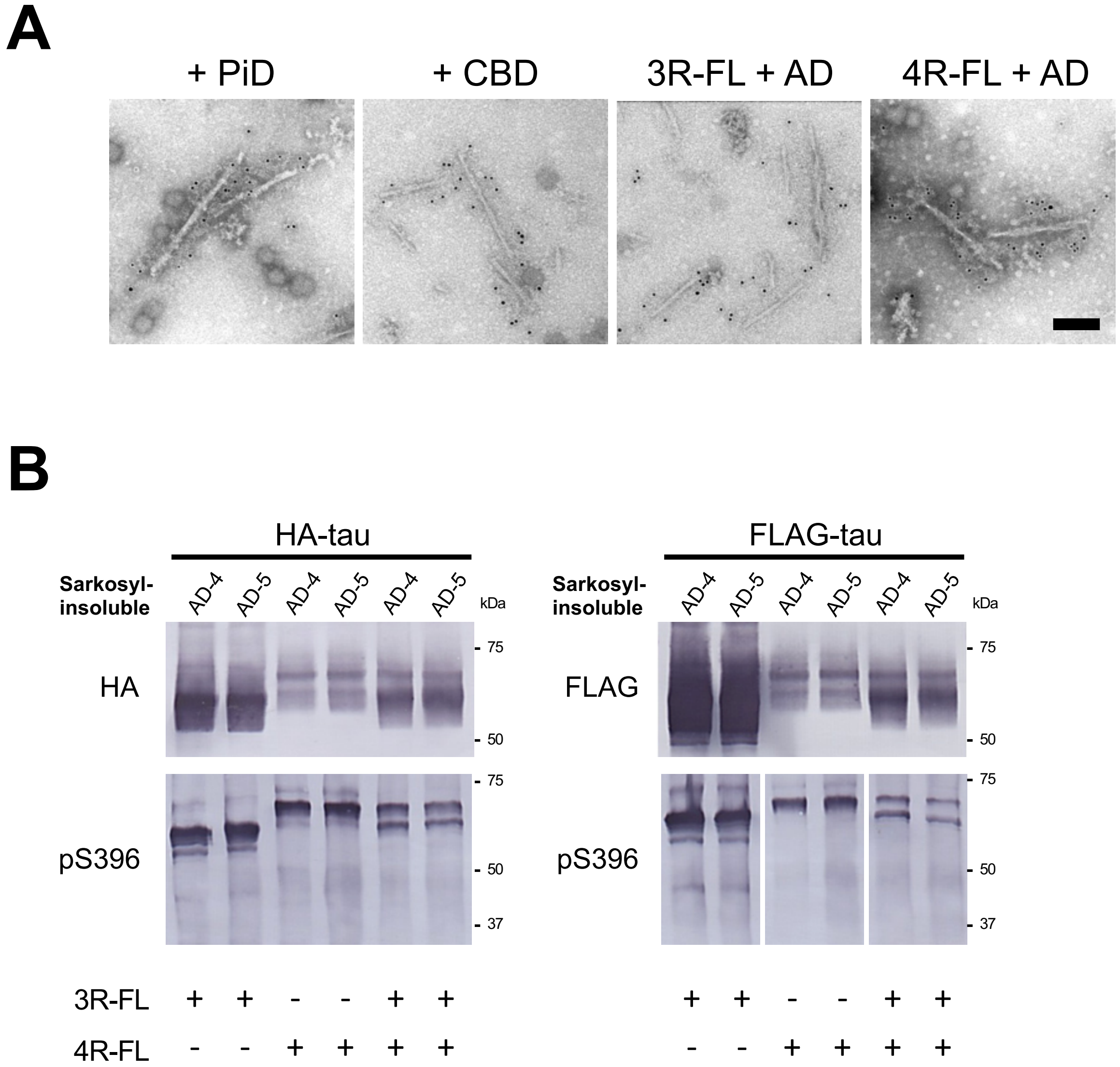

Supplemental Fig 8. Electron microscopic analysis of insoluble fractions extracted from transfected cells with patient-derived tau strains

A, Immunoelectron microscopy of sarkosyl-insoluble fractions extracted from transfected cells transiently expressing HA-tagged human tau 3R1N or 4R1N with PiD-, CBD- and AD-tau seeds. Electron micrographs show fibrous structures positive for anti-HA antibody, labeled with secondary antibody conjugated to 6 nm gold particles. Scale bar, 100 nm.

B, Sarkosyl-insoluble fractions prepared from AD-4 and AD-5 cases (1 μl) were introduced into SH-SY5Y cells transiently expressing HA-tagged human tau 3R1N and 4R1N (left) or FLAG-tagged human tau 3R1N and 4R1N (right). Immunoblot analysis of sarkosyl-insoluble fractions extracted from cells transfected with sarkosyl-insoluble fractions from AD-4 and AD-5 cases. Insoluble tau was detected with pS396, anti-HA and anti-FLAG antibodies. Full-length blots are presented in Supplementary material.

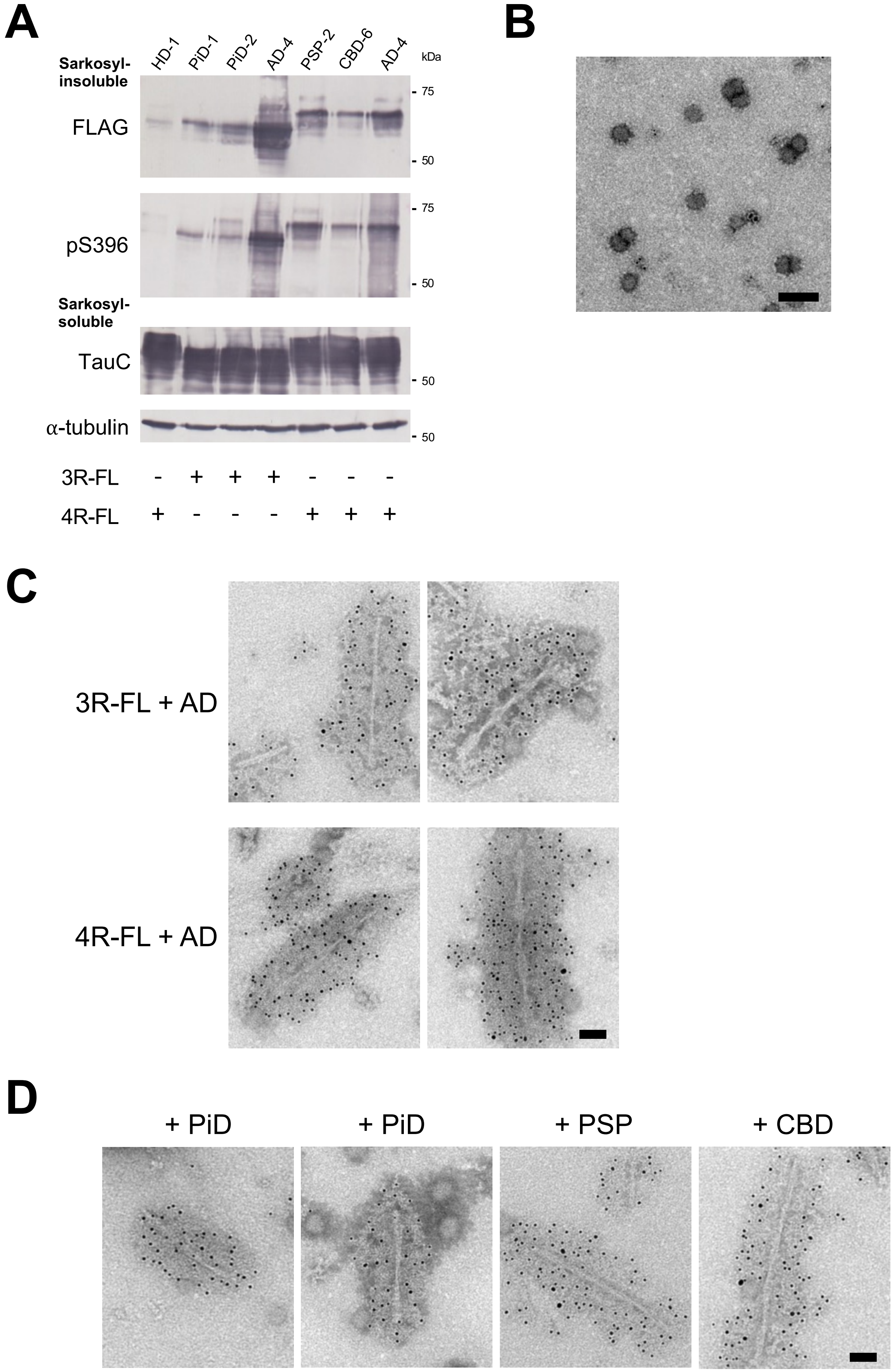

A
HD-1
PiD-1
PiD-2
AD-4
PSP-2
CBD-6
AD-4
kDa
Sarkosyl-insoluble
FLAG
75
50
pS396
75
50
Sarkosyl-soluble
TauC
50
α-tubulin
50
3R-FL - + + + - - -
4R-FL + - - - + + +
B
C
3R-FL + AD
4R-FL + AD
D
+ PiD
+ PiD
+ PSP
+ CBD

Supplemental Fig 9. Electron microscopic analysis of insoluble fractions extracted from transfected cells expressing FLAG-tagged tau with patient-derived tau strains

A, Sarkosyl-insoluble fractions prepared from PiD, PSP, CBD and AD cases were introduced into SH-SY5Y cells transiently expressing FLAG-tagged human tau 3R1N or 4R1N. Immunoblot analysis of sarkosyl-insoluble fractions and sarkosyl-soluble fractions extracted from transfected cells with sarkosyl-insoluble fractions from the PiD-1, PiD-2, PSP-2, CBD-6 and AD-4 cases. Insoluble tau was detected with anti-FLAG and pS396 antibodies. Total tau was detected with TauC antibody. Full-length blots are presented in Supplementary material.

B, Immunoelectron microscopy of sarkosyl-insoluble fractions extracted from transfected cells with introduced HD seeds. Scale bar, 100 nm.

C, Immunoelectron microscopy of sarkosyl-insoluble fractions extracted from transfected cells expressing FLAG-tagged human tau 3R1N (upper) and 4R1N (lower) with AD-tau seeds. Electron micrographs show fibrous structures positive for anti-FLAG antibody, labeled with secondary antibody conjugated to 5 nm gold particles. Scale bar, 50 nm.

D, Immunoelectron microscopy of sarkosyl-insoluble fractions extracted from transfected cells with PiD-, PSP- and CBD-tau seeds. Electron micrographs show fibrous structures positive for anti-FLAG antibody, labeled with secondary antibody conjugated to 5 nm gold particles. Scale bar, 50 nm.
