# Supplementary material for: Human tauopathy-derived tau strains determine the substrates recruited for templated amplification
Source: Brain. 2021 Mar 9;144(8):2333–48. doi: 10.1093/brain/awab091 (PMC8418341; doi:10.1093/brain/awab091)

Source images of immunoblots

Fig.1A

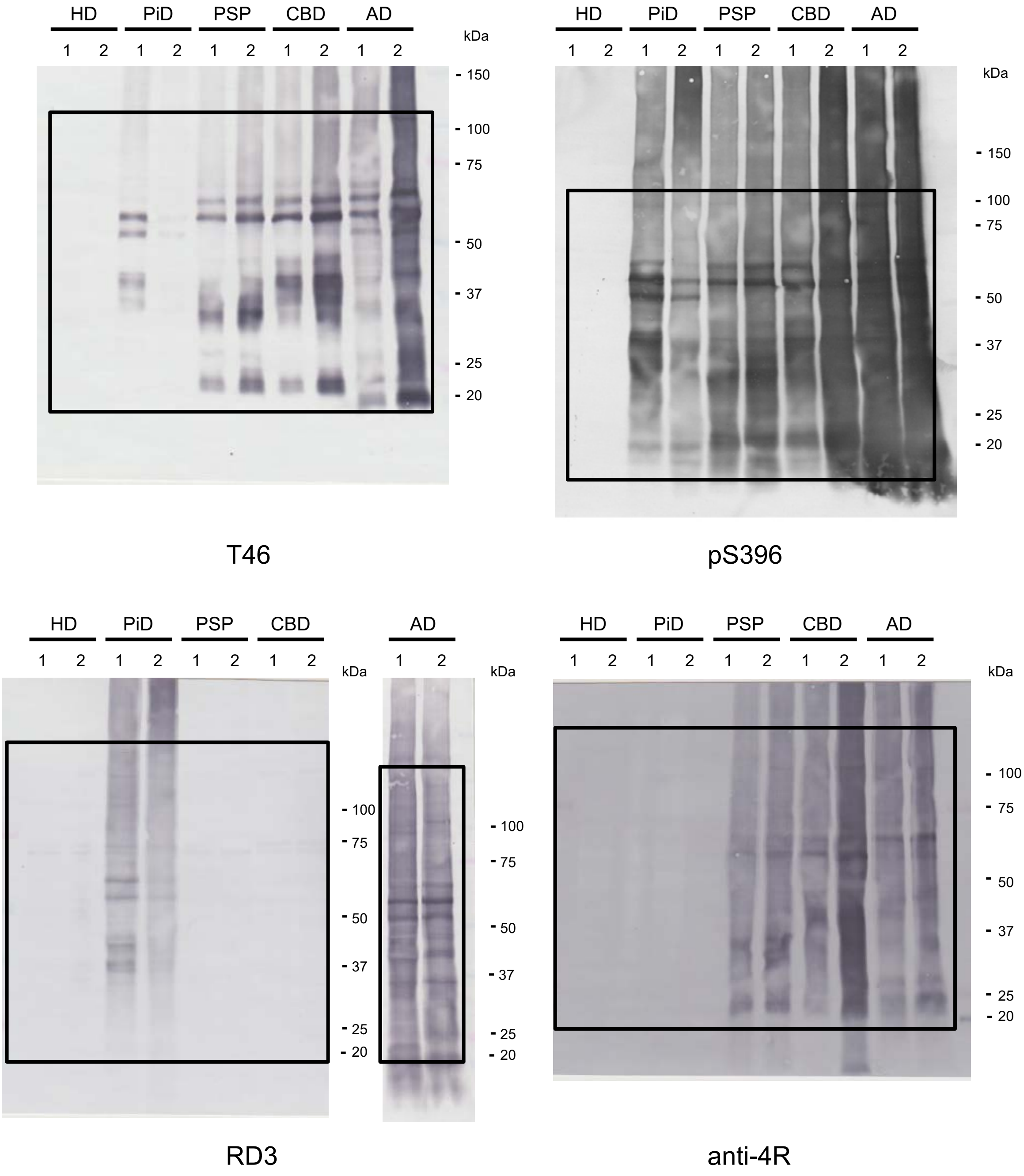

Source images of immunoblots

Fig.2A

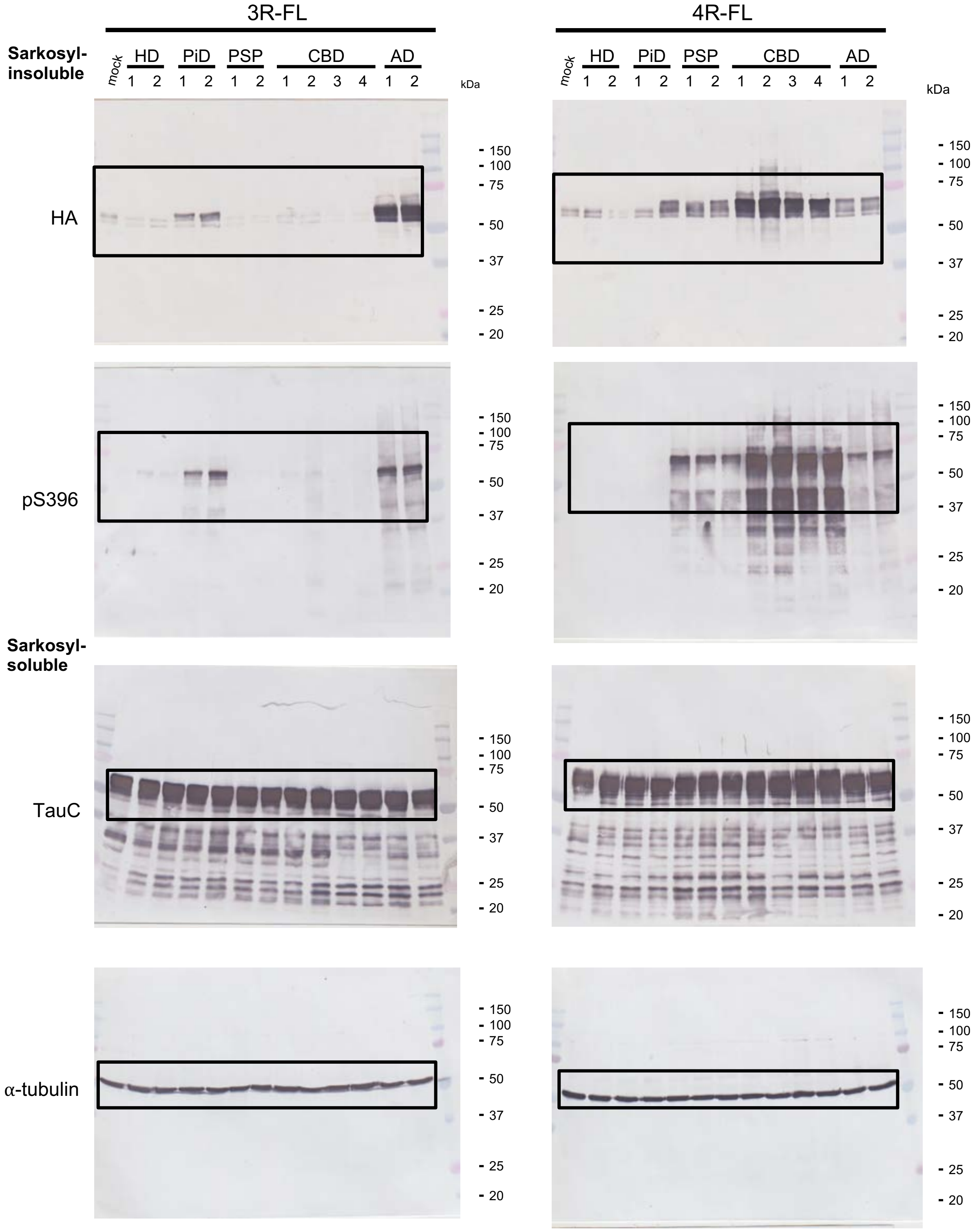

Source images of immunoblots

Fig.2C

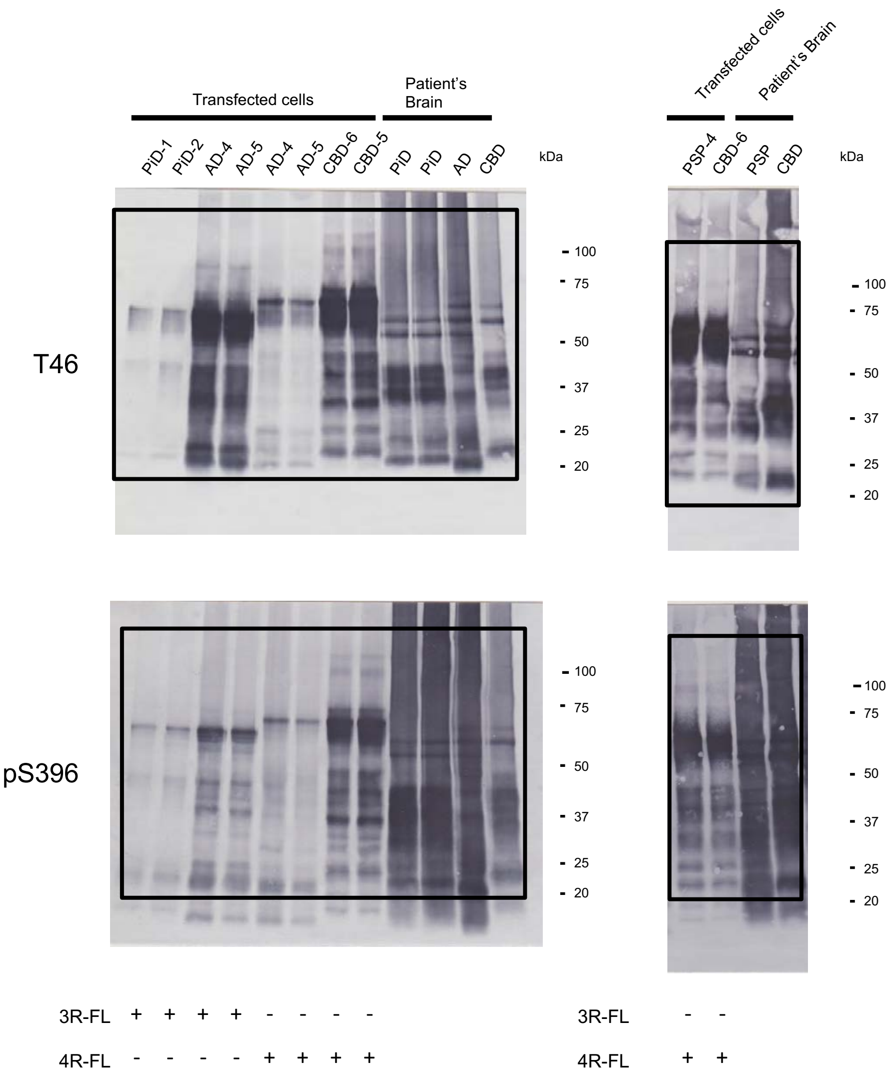

Source images of immunoblots

Fig.3A

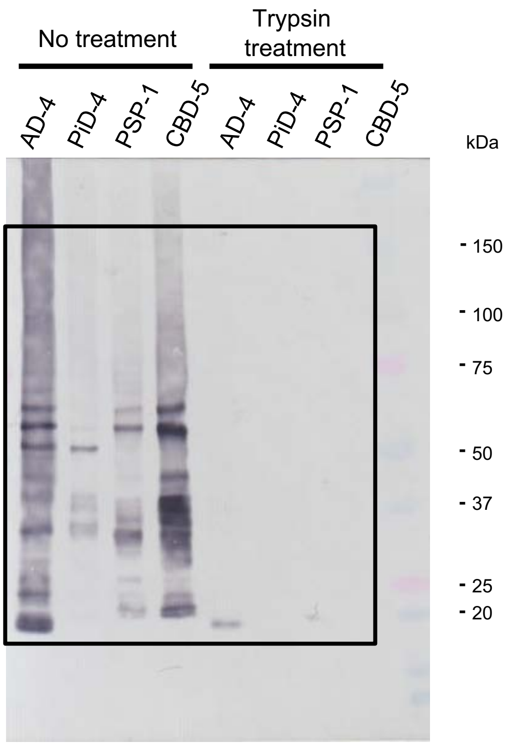

T46

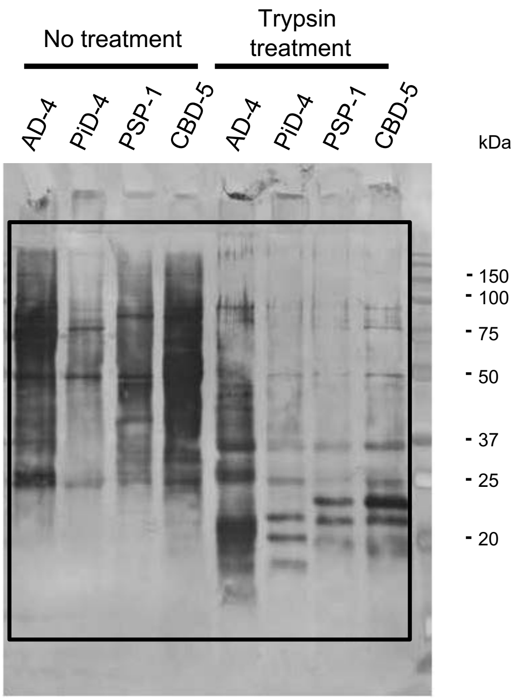

Tau 360-380

Source images of immunoblots

Fig.3B

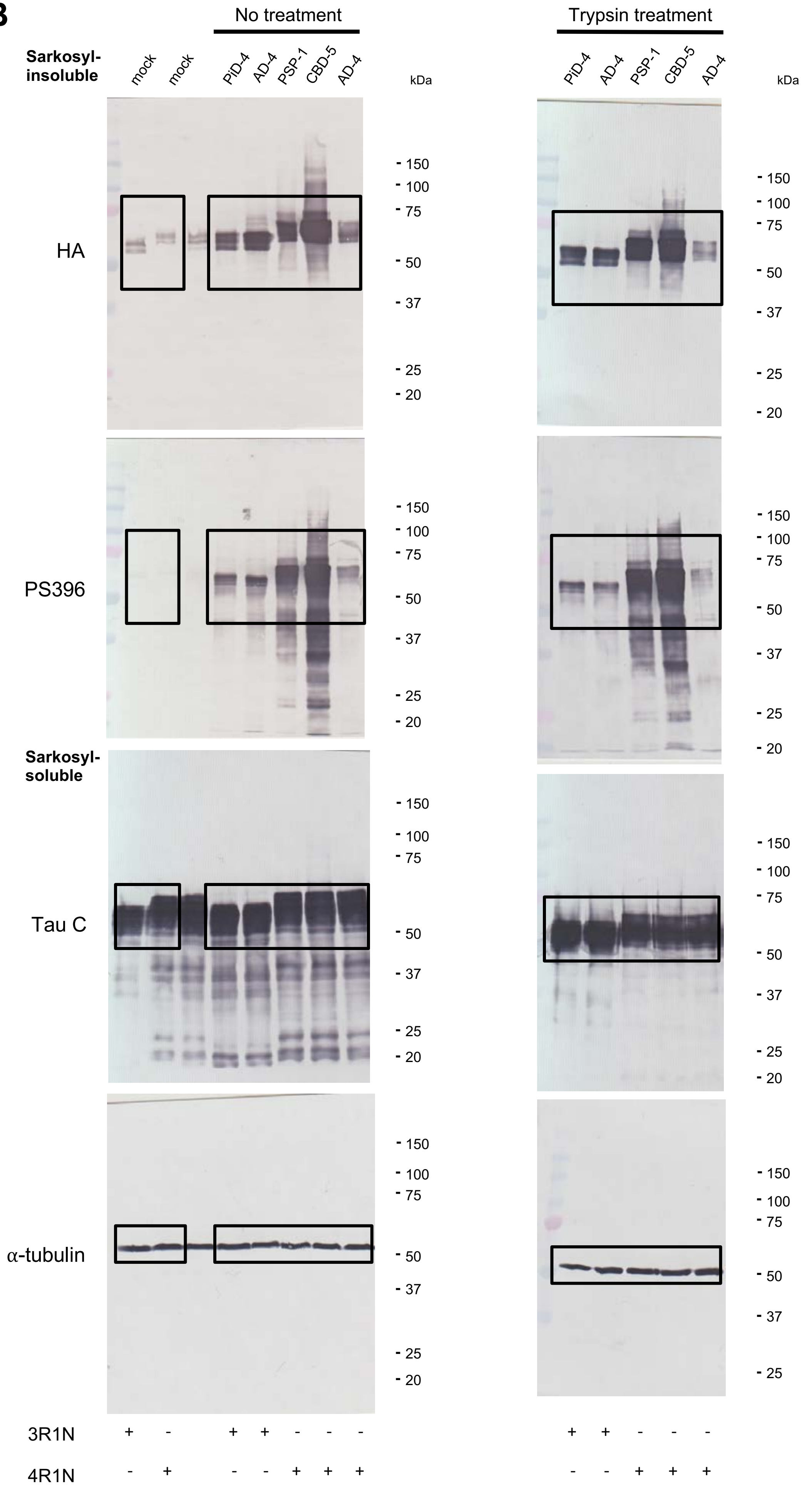

Source images of immunoblots

Fig.4A

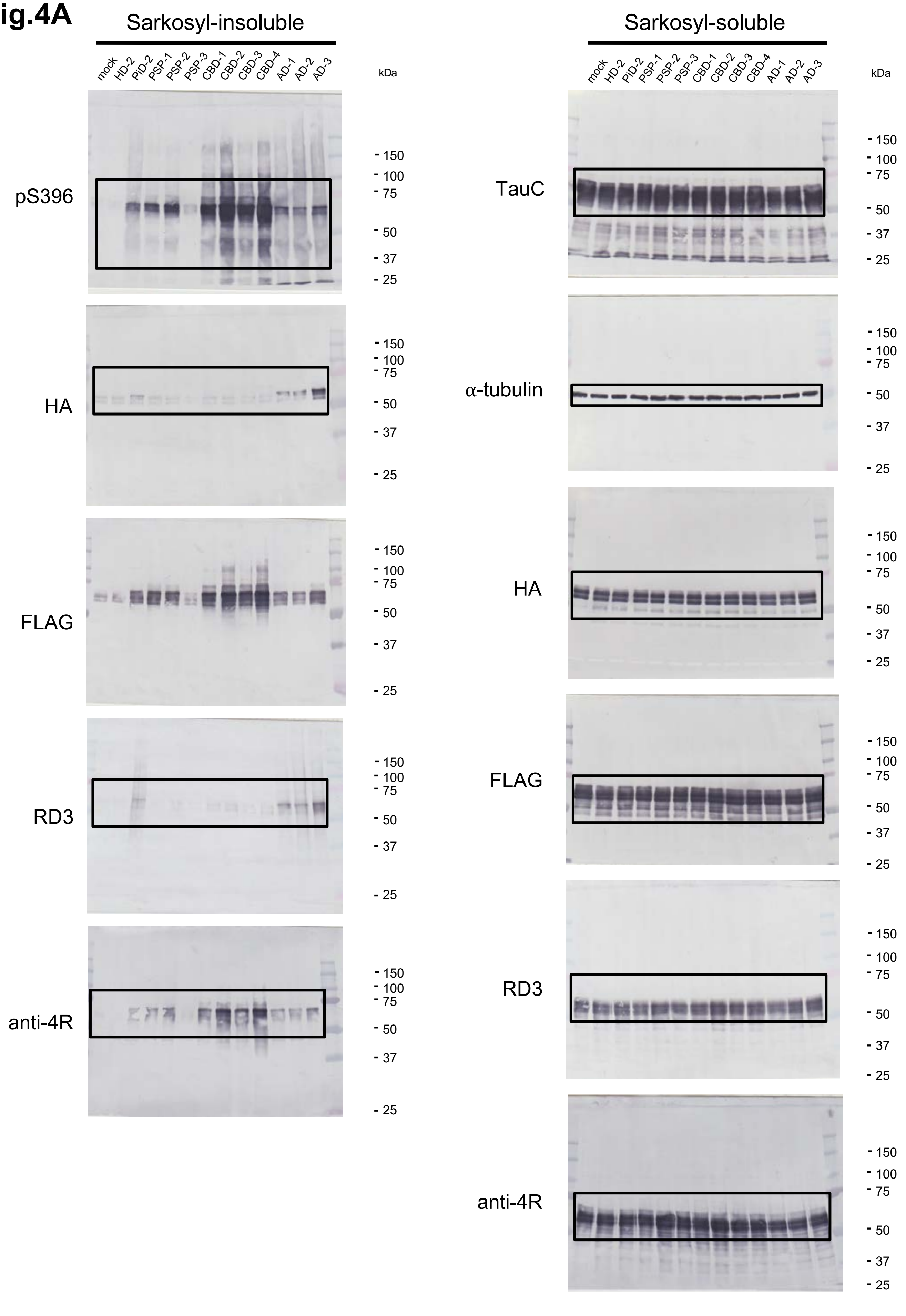

Source images of immunoblots

Fig.5E

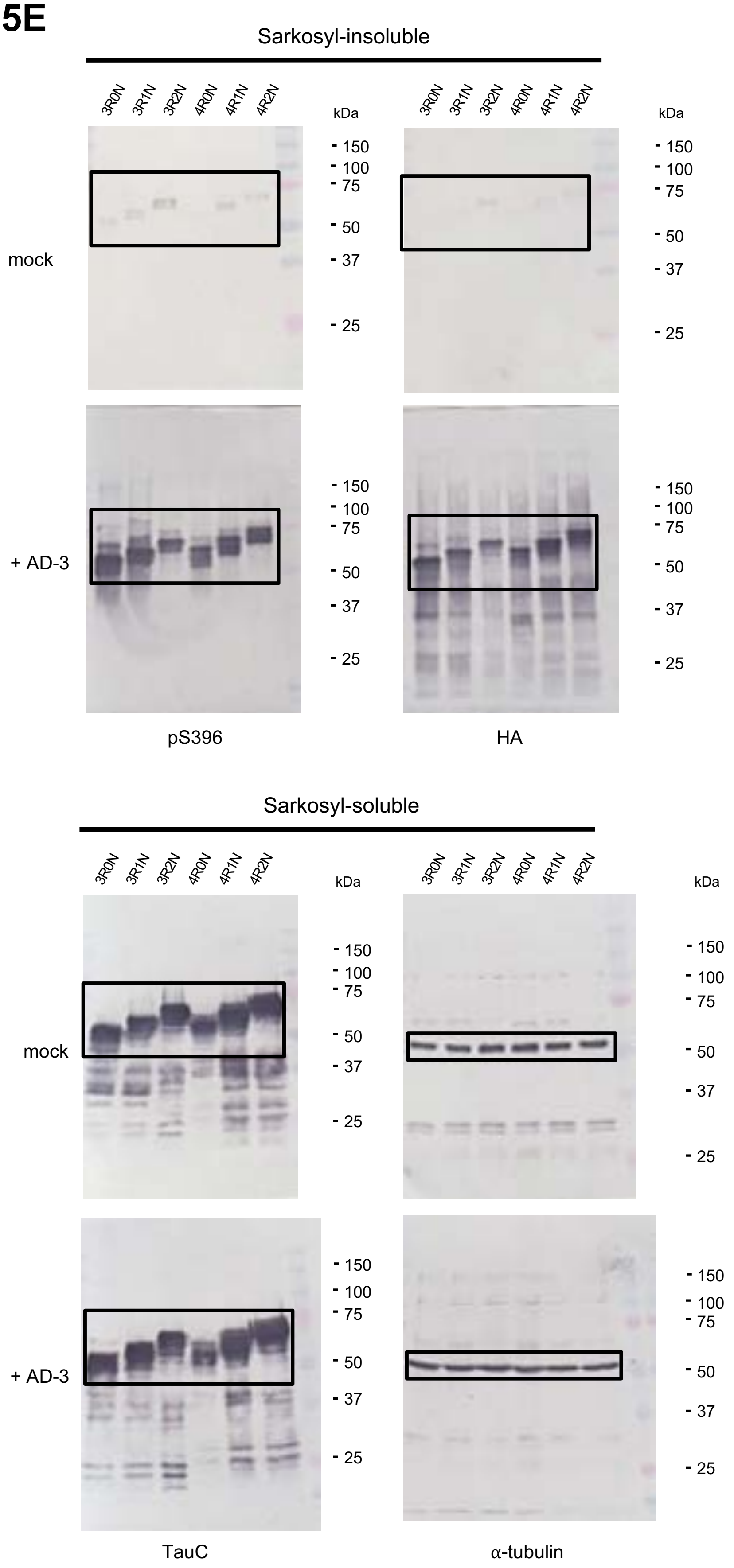

Source images of immunoblots

Fig.7A

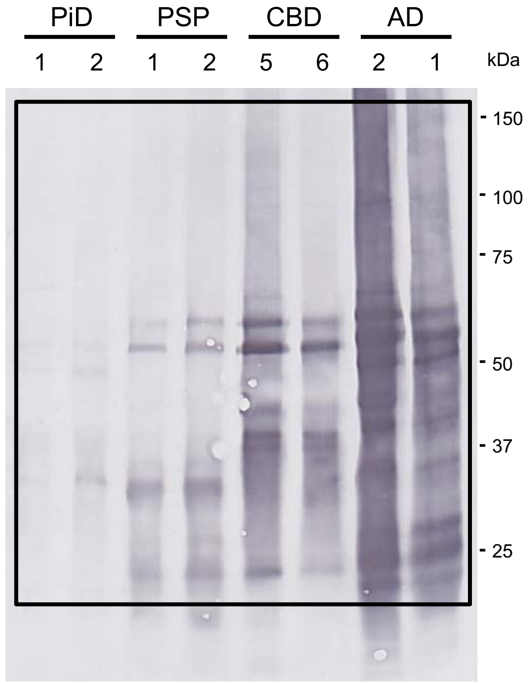

Source images of immunoblots

Fig.7C

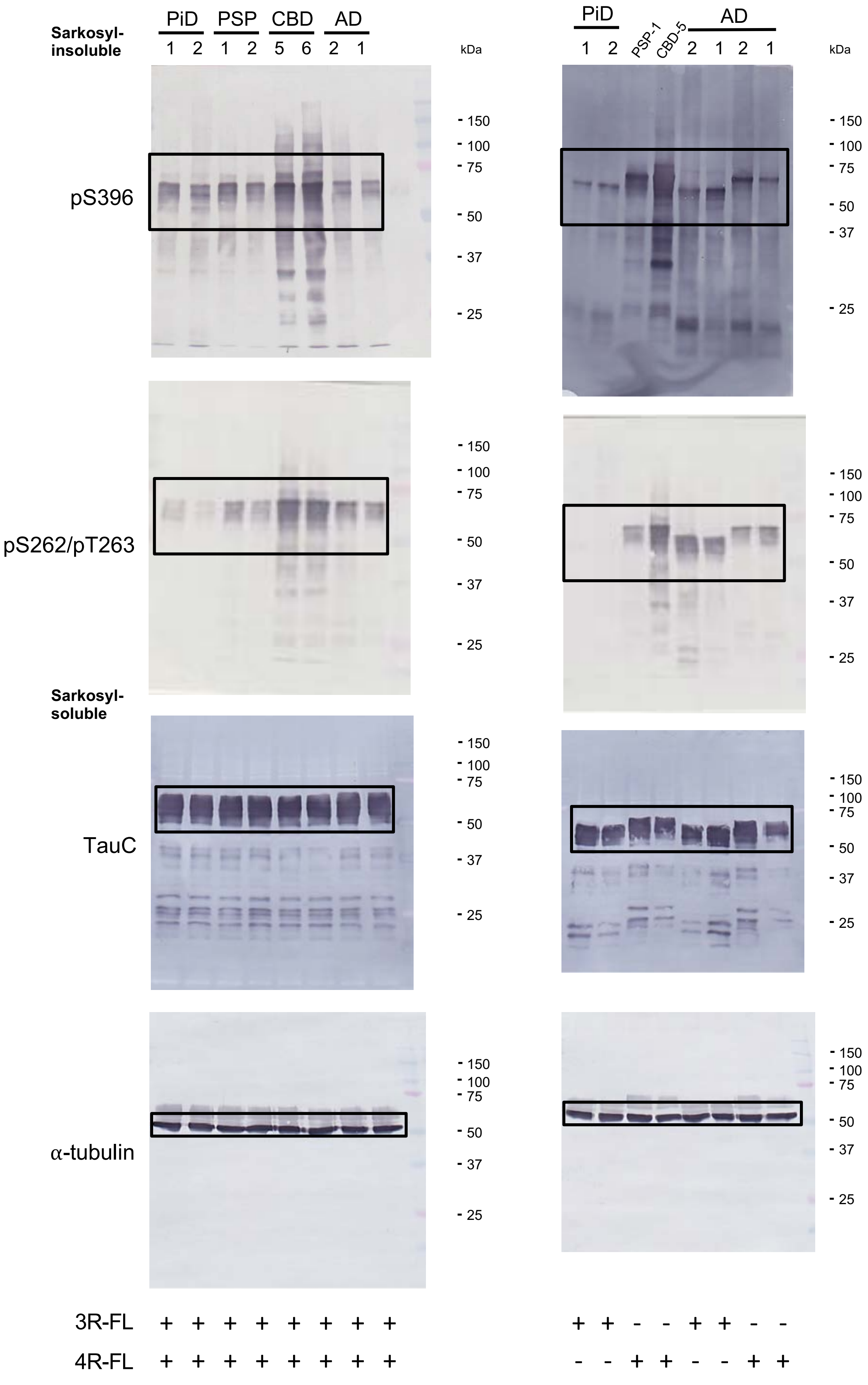

Source images of immunoblots

Fig.8A

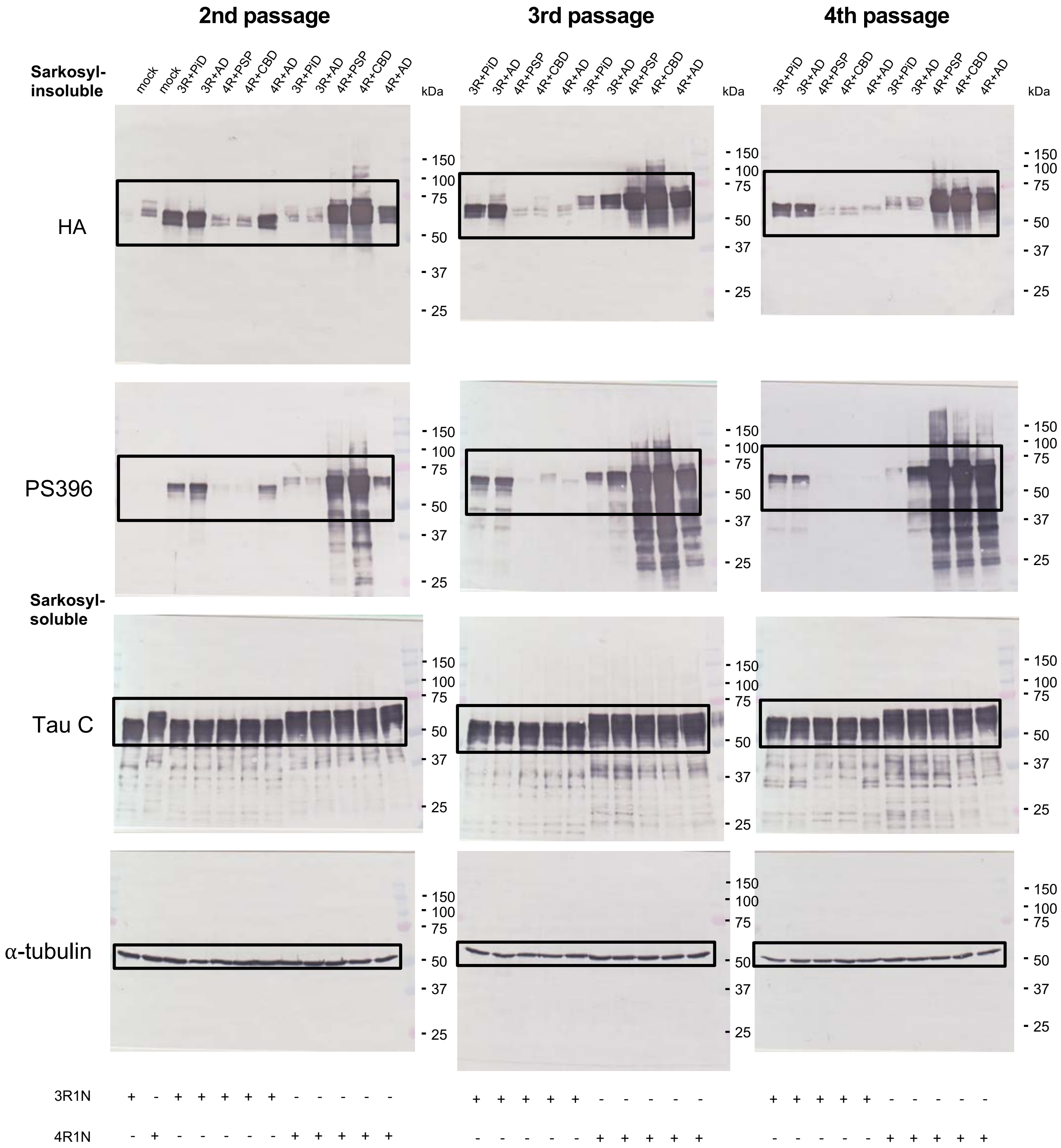

Source images of immunoblots

Supplemental Fig. 2

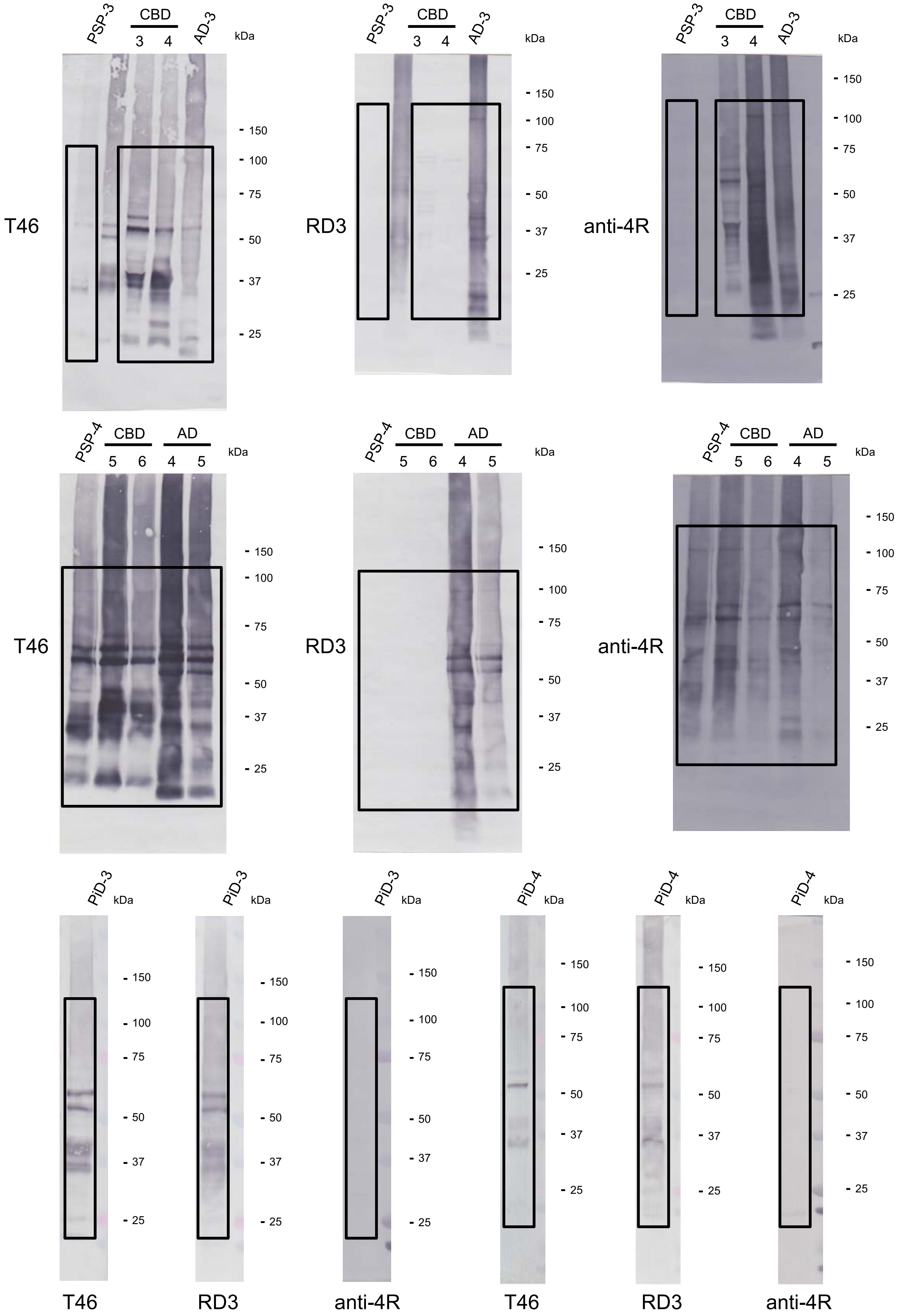

Source images of immunoblots

Supplemental Fig. 3A

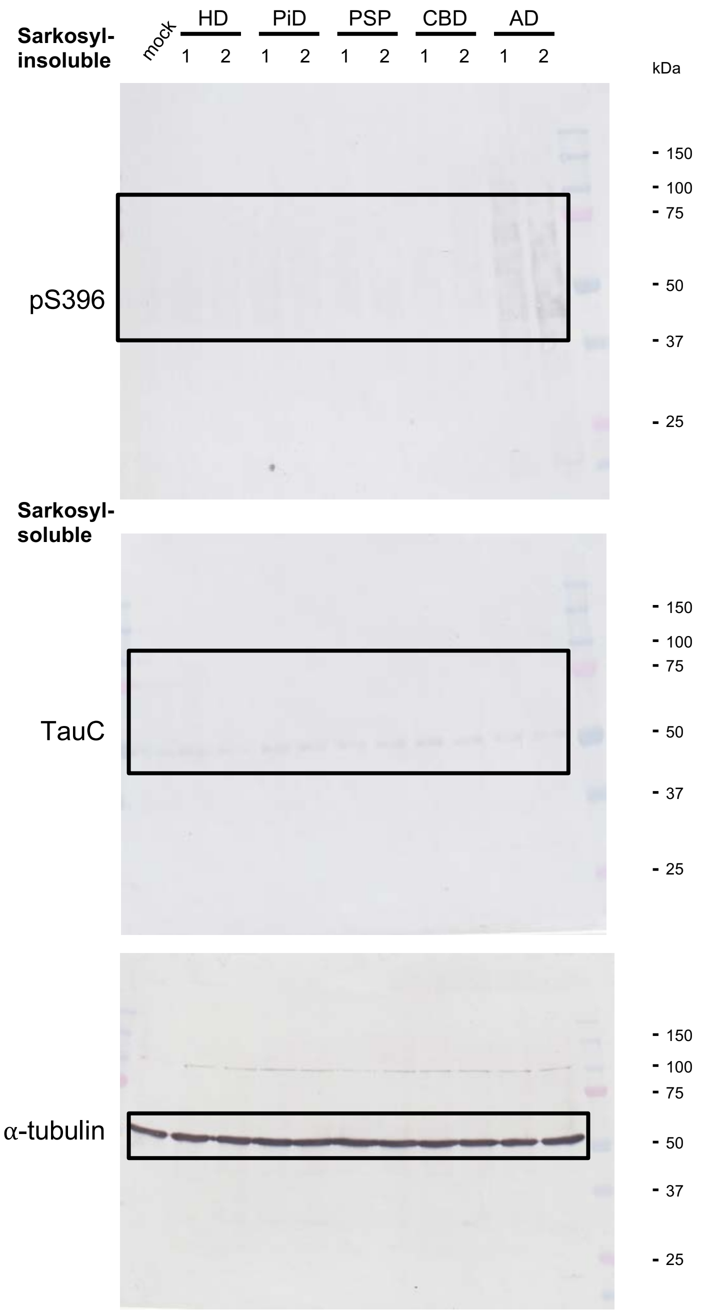

Source images of immunoblots

Supplemental Fig. 3B

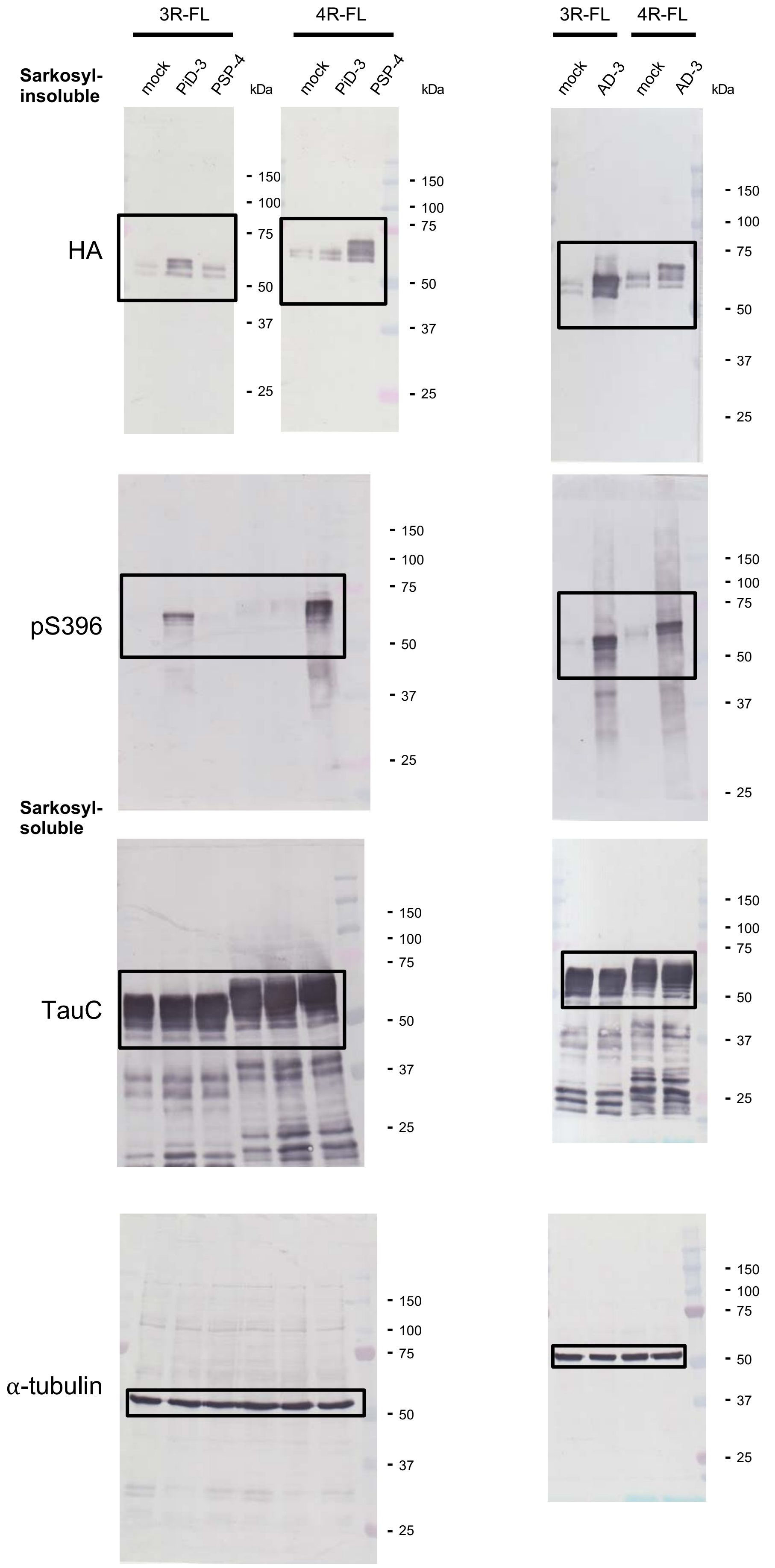

Source images of immunoblots

Supplemental Fig. 3C

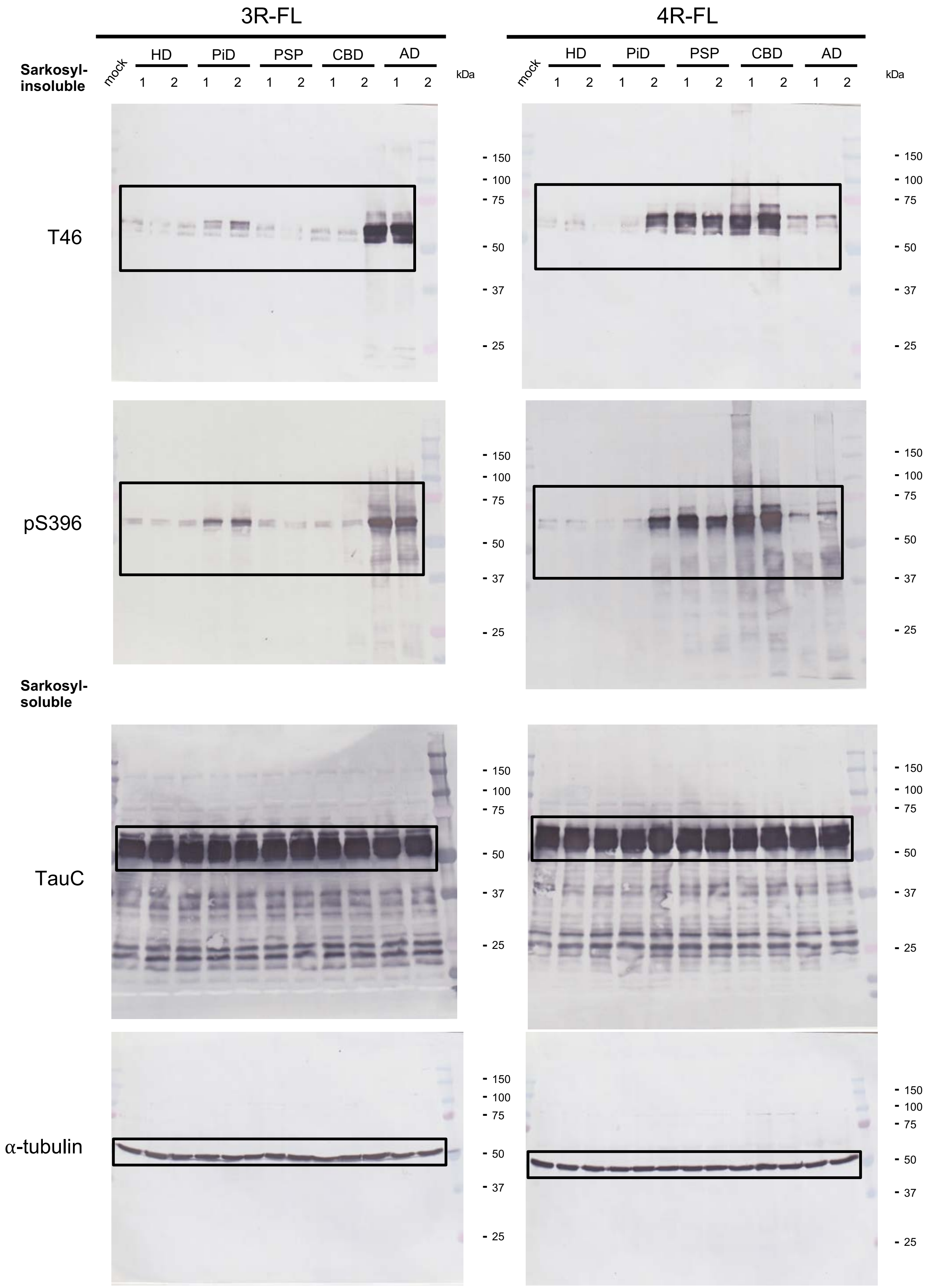

Source images of immunoblots

Supplemental Fig. 3D

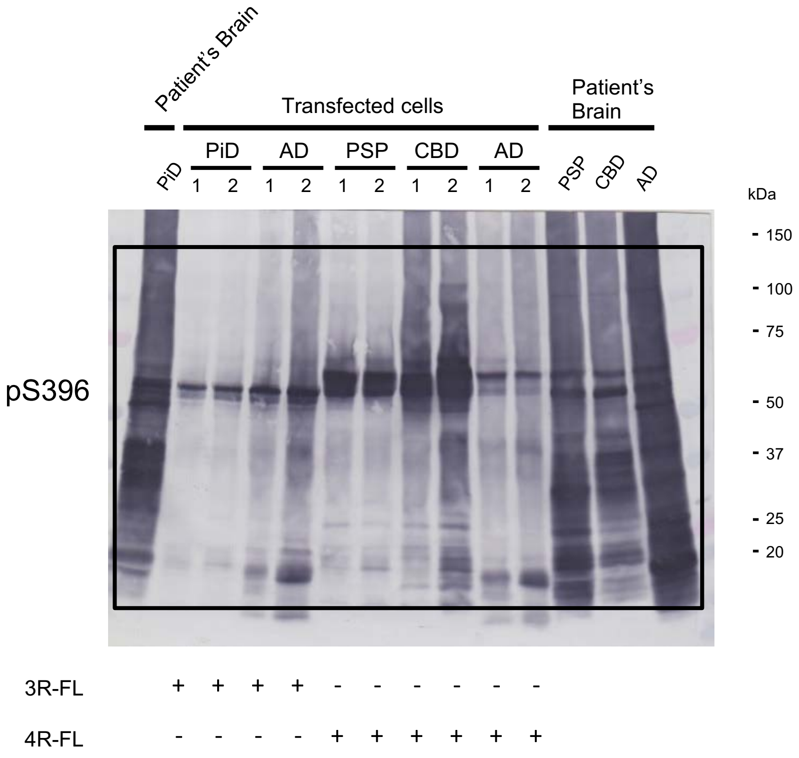

Source images of immunoblots

Supplemental Fig. 4A

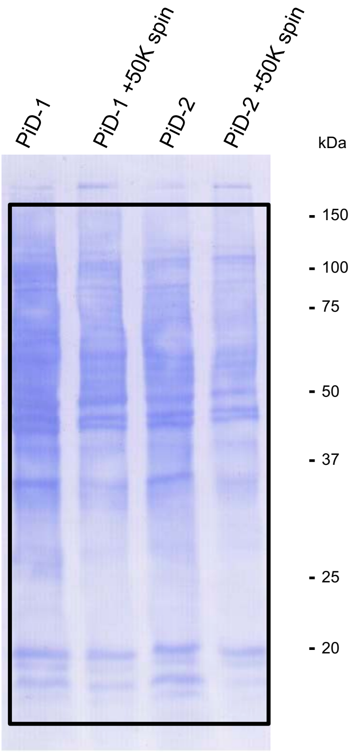

Supplemental Fig. 4B

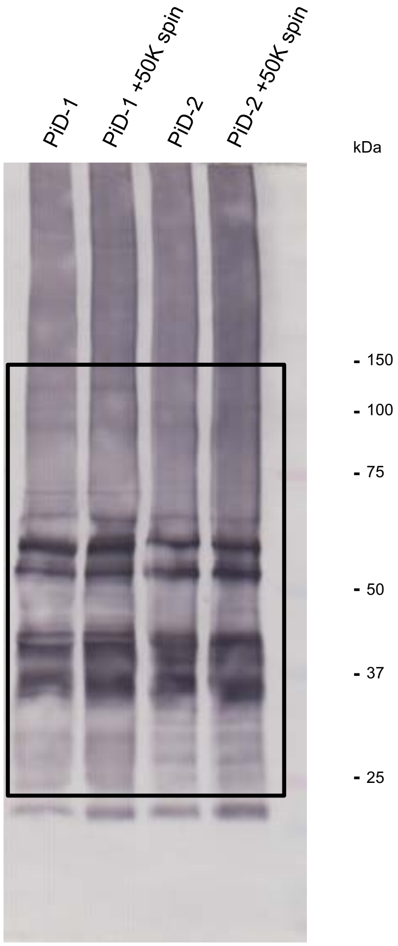

T46

Source images of immunoblots

Supplemental Fig. 4C

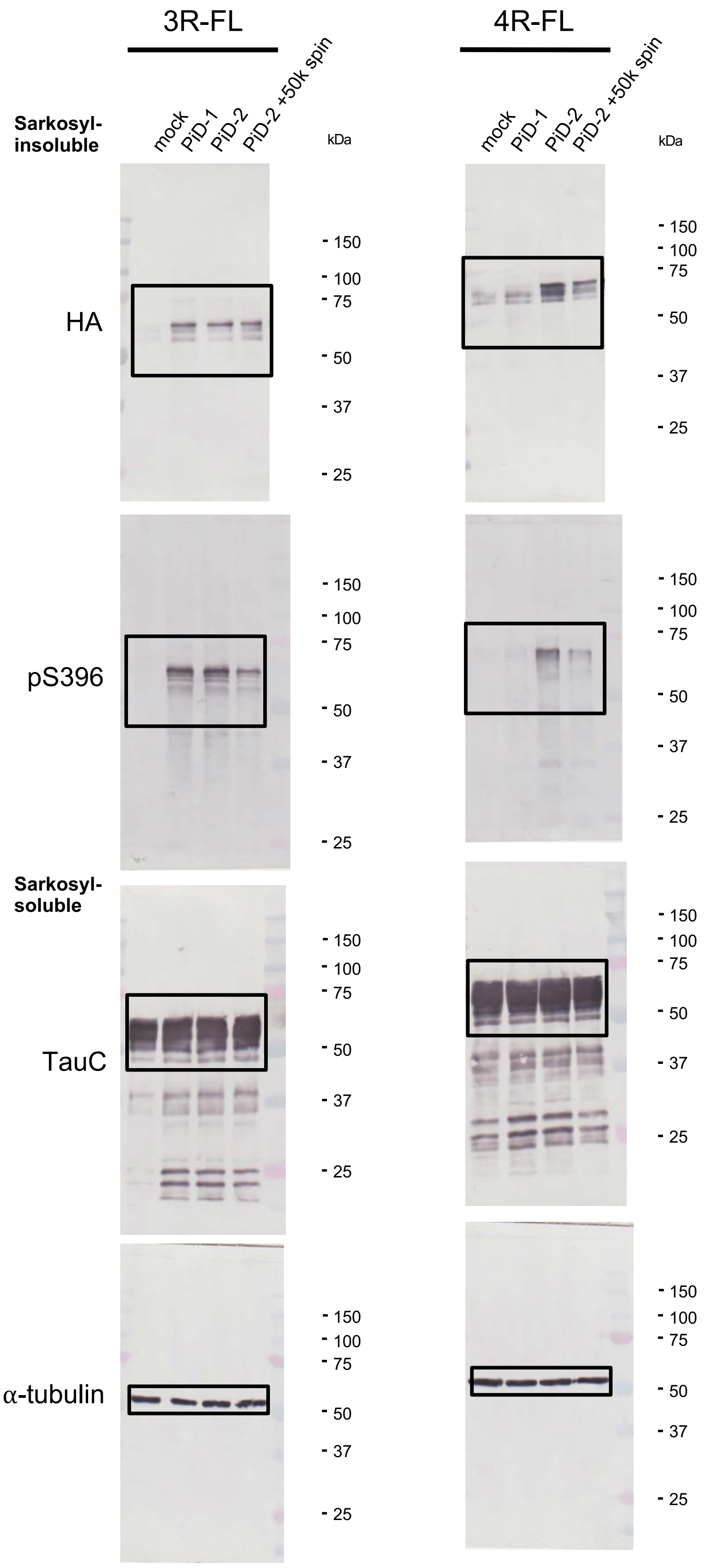

Source images of immunoblots

Supplemental Fig. 4E

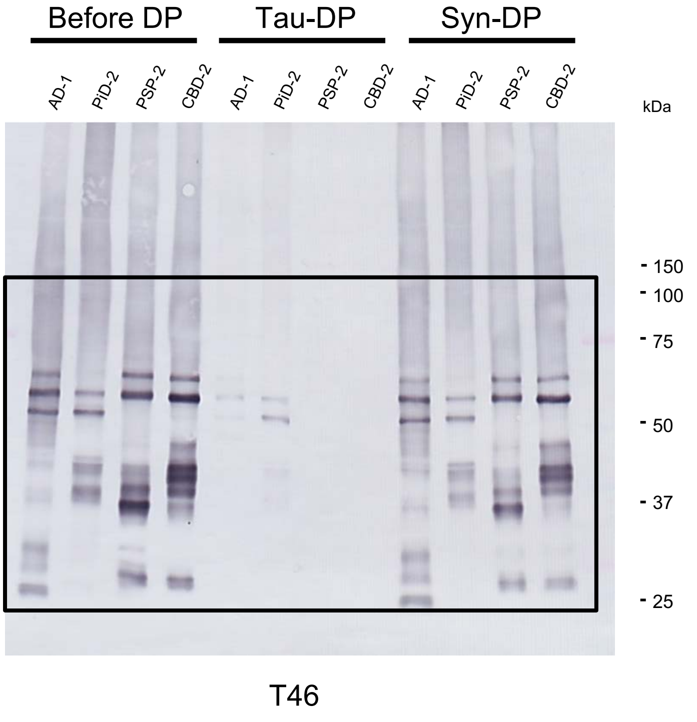

Source images of immunoblots

Supplemental Fig. 4E

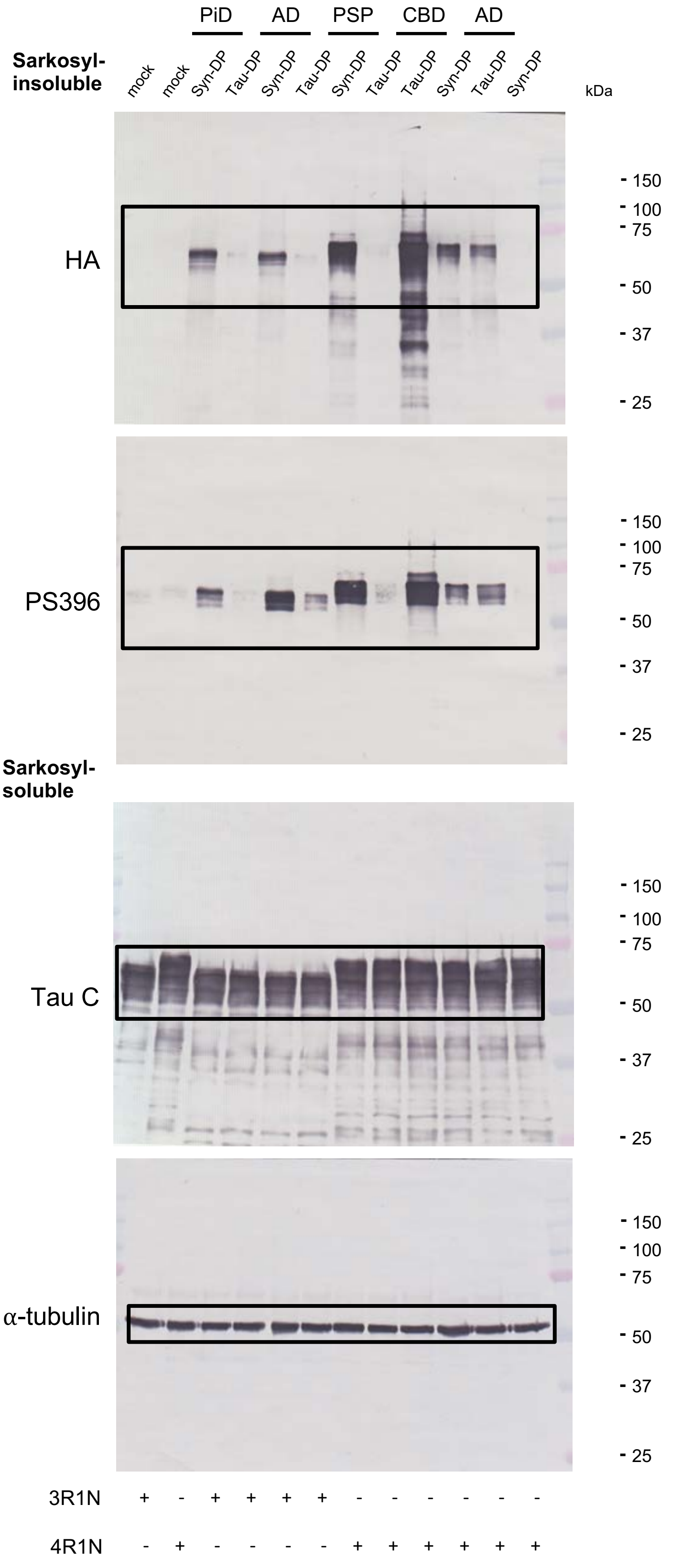

Source images of immunoblots

Supplemental Fig. 6

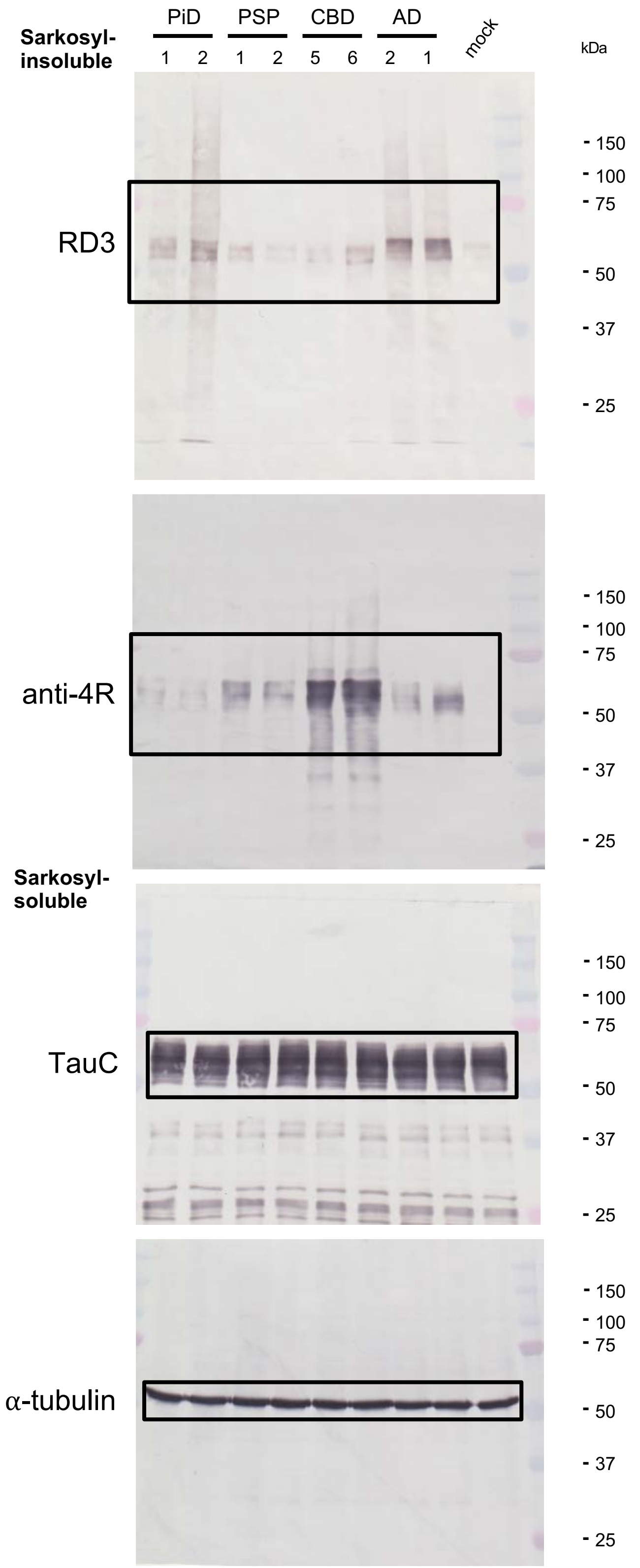

Source images of immunoblots

Supplemental Fig. 7A

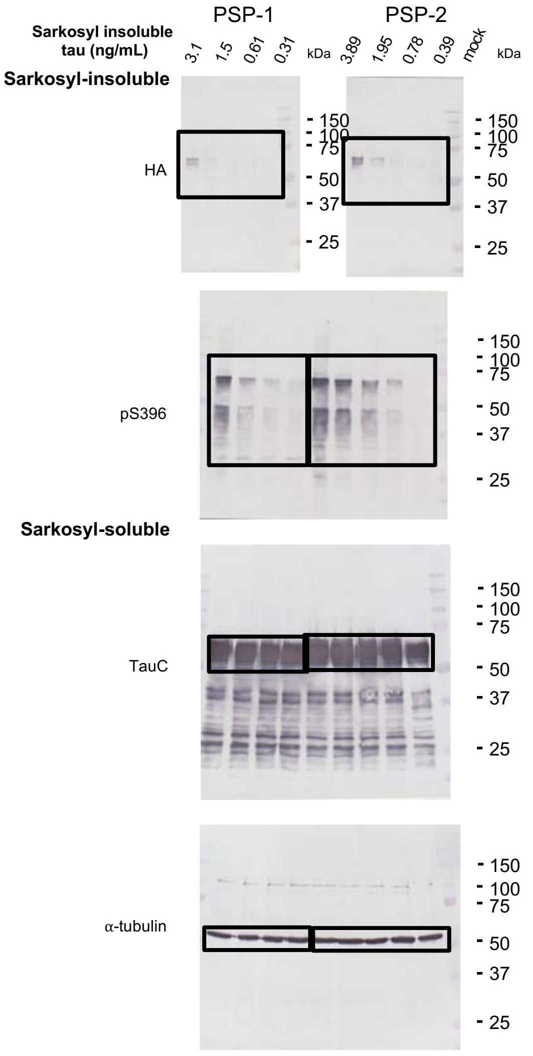

Supplemental Fig. 7B

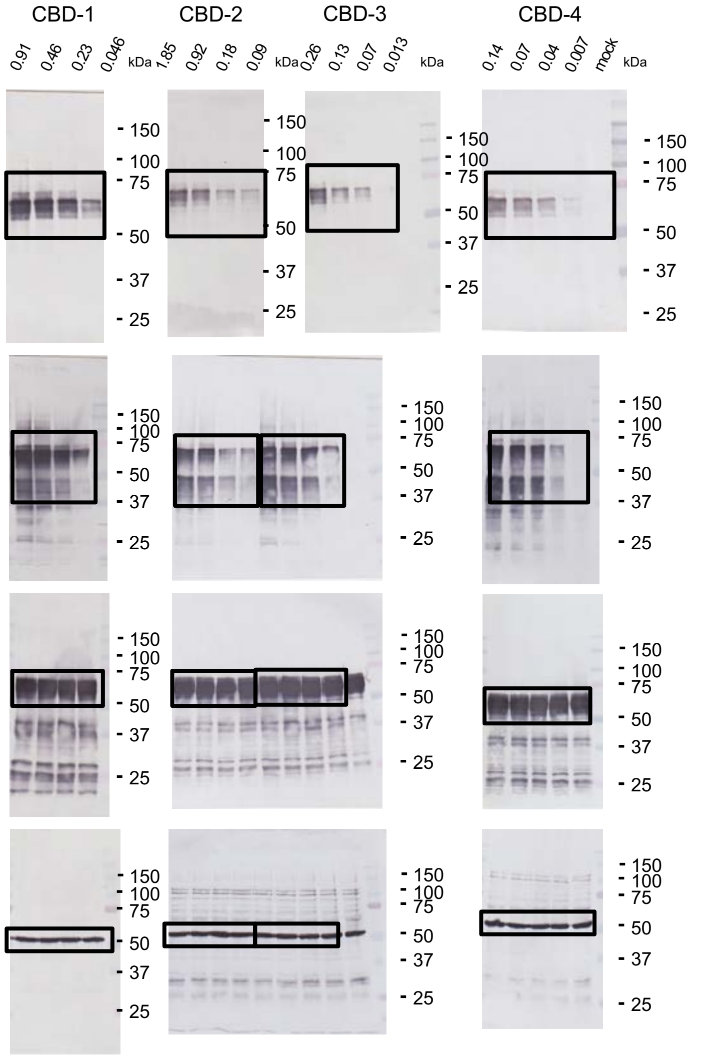

Source images of immunoblots

Supplemental Fig. 7C

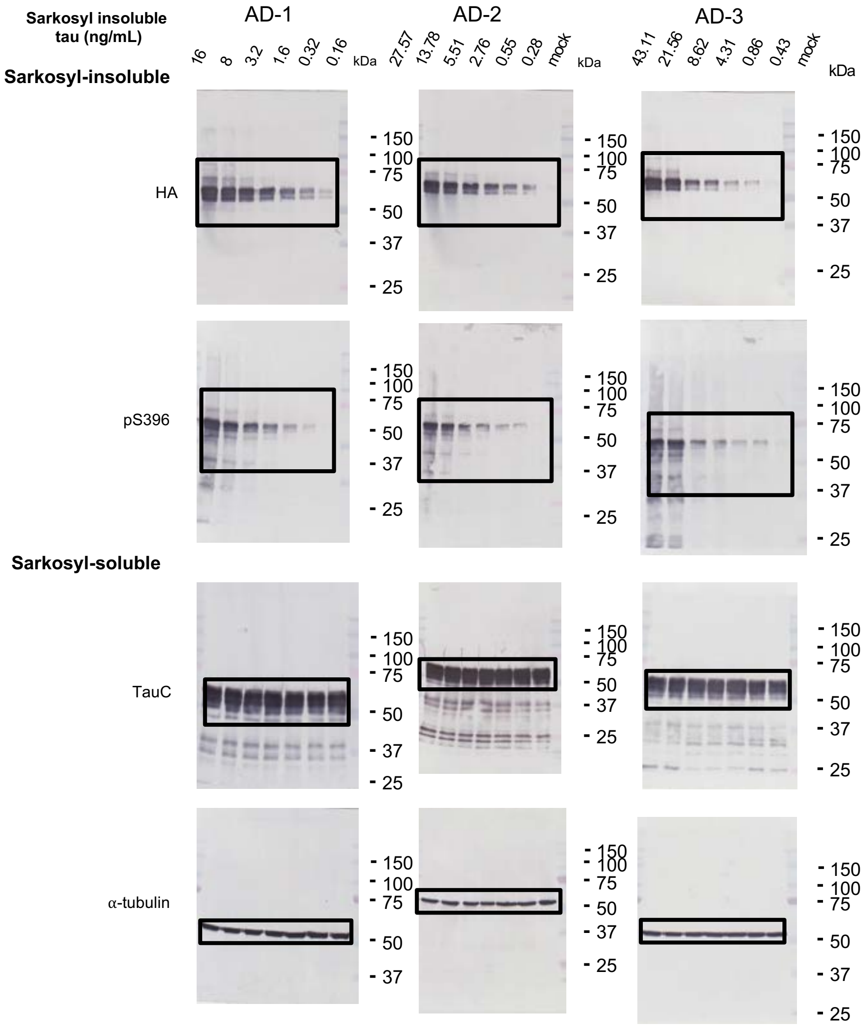

Source images of immunoblots

Supplemental Fig. 7D

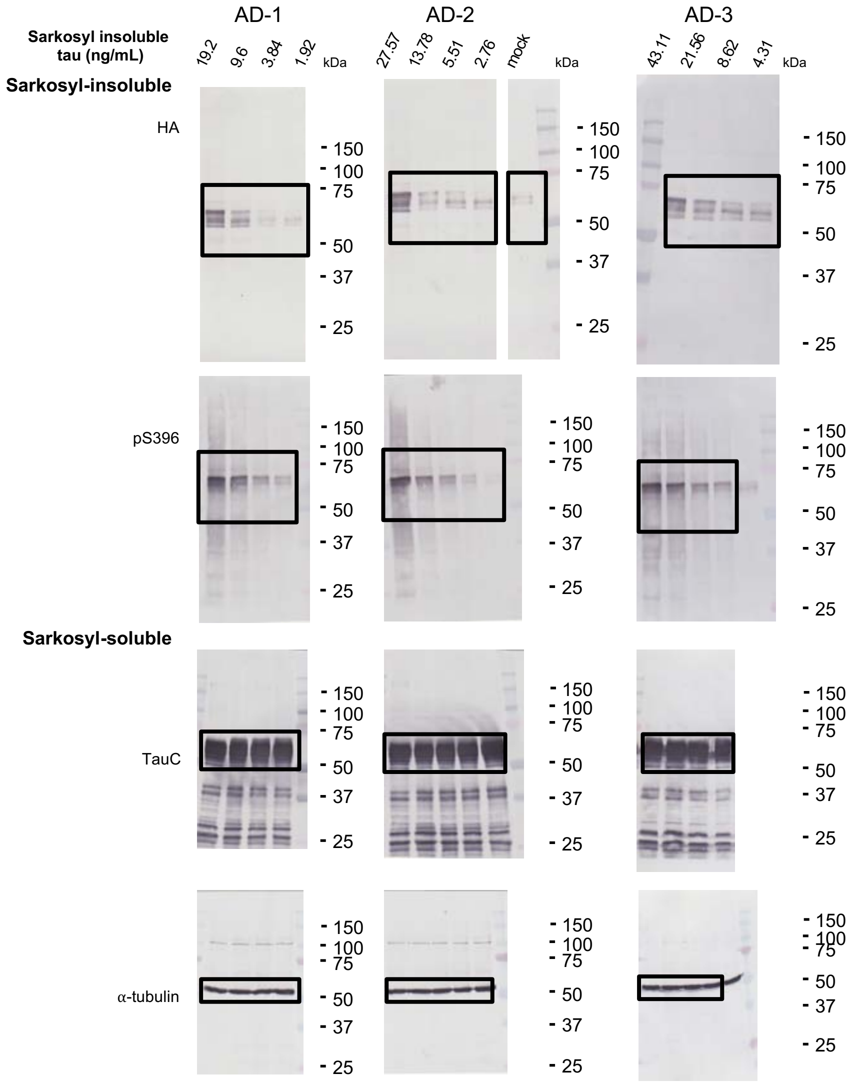

Source images of immunoblots

Supplemental Fig. 8A

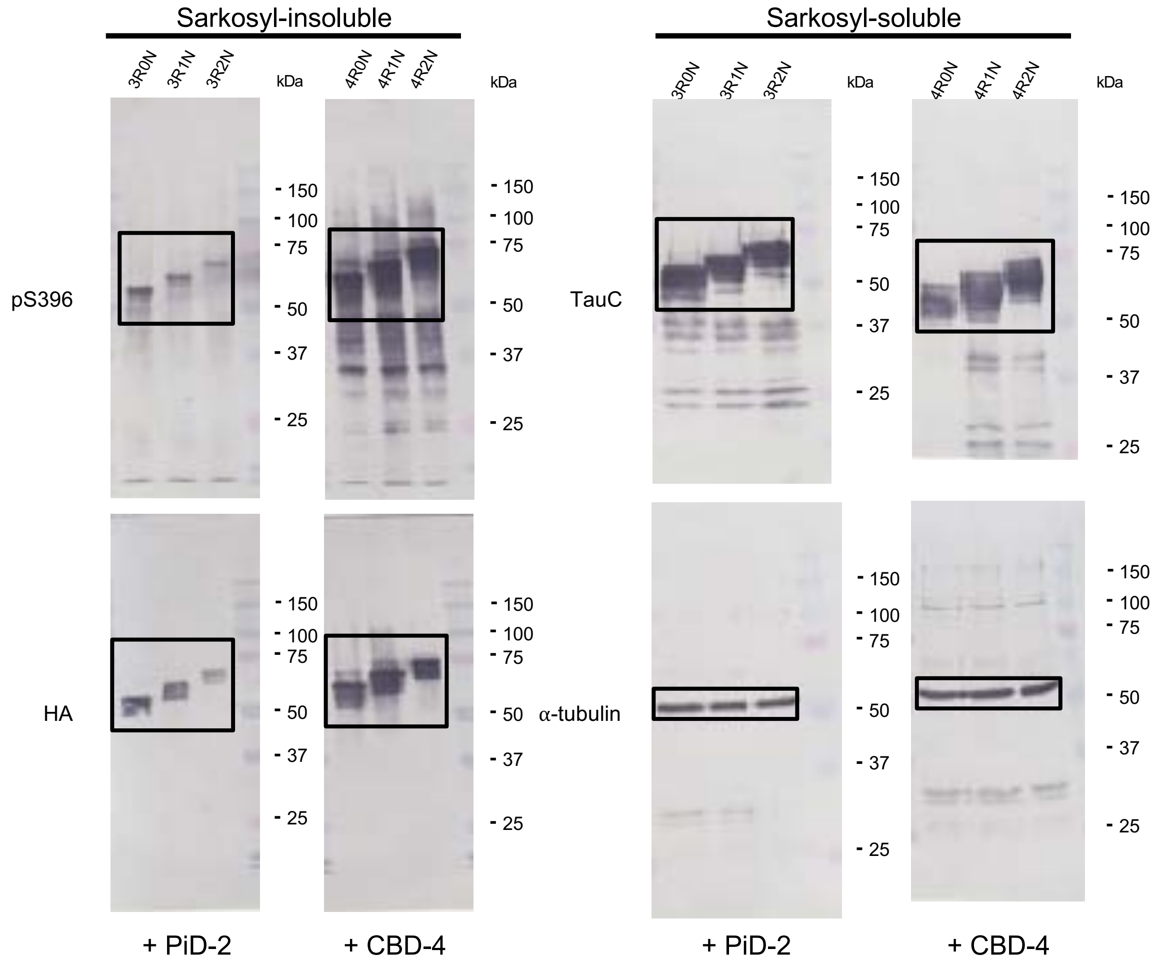

Supplemental Fig. 8C

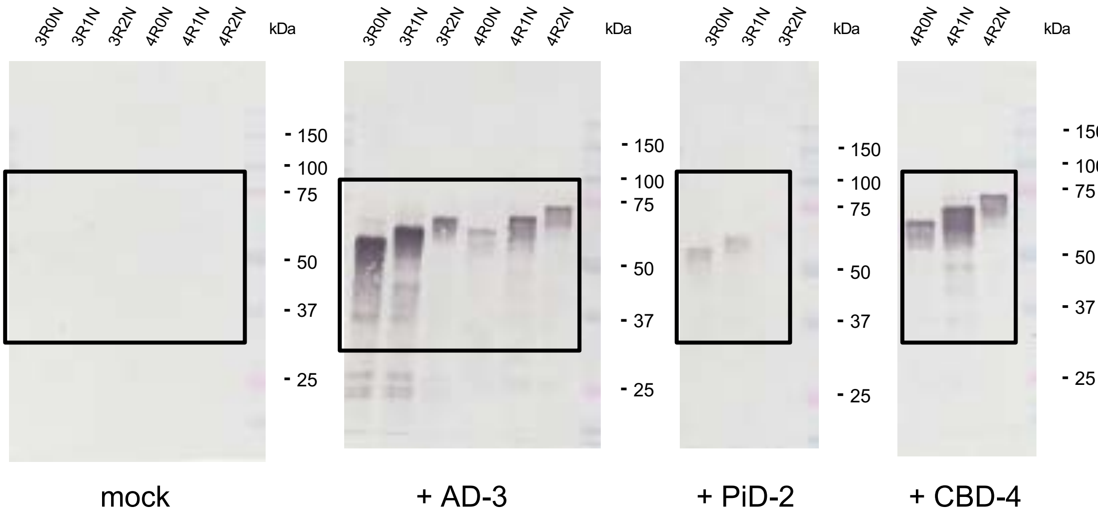

Source images of immunoblots

Supplemental Fig. 9B

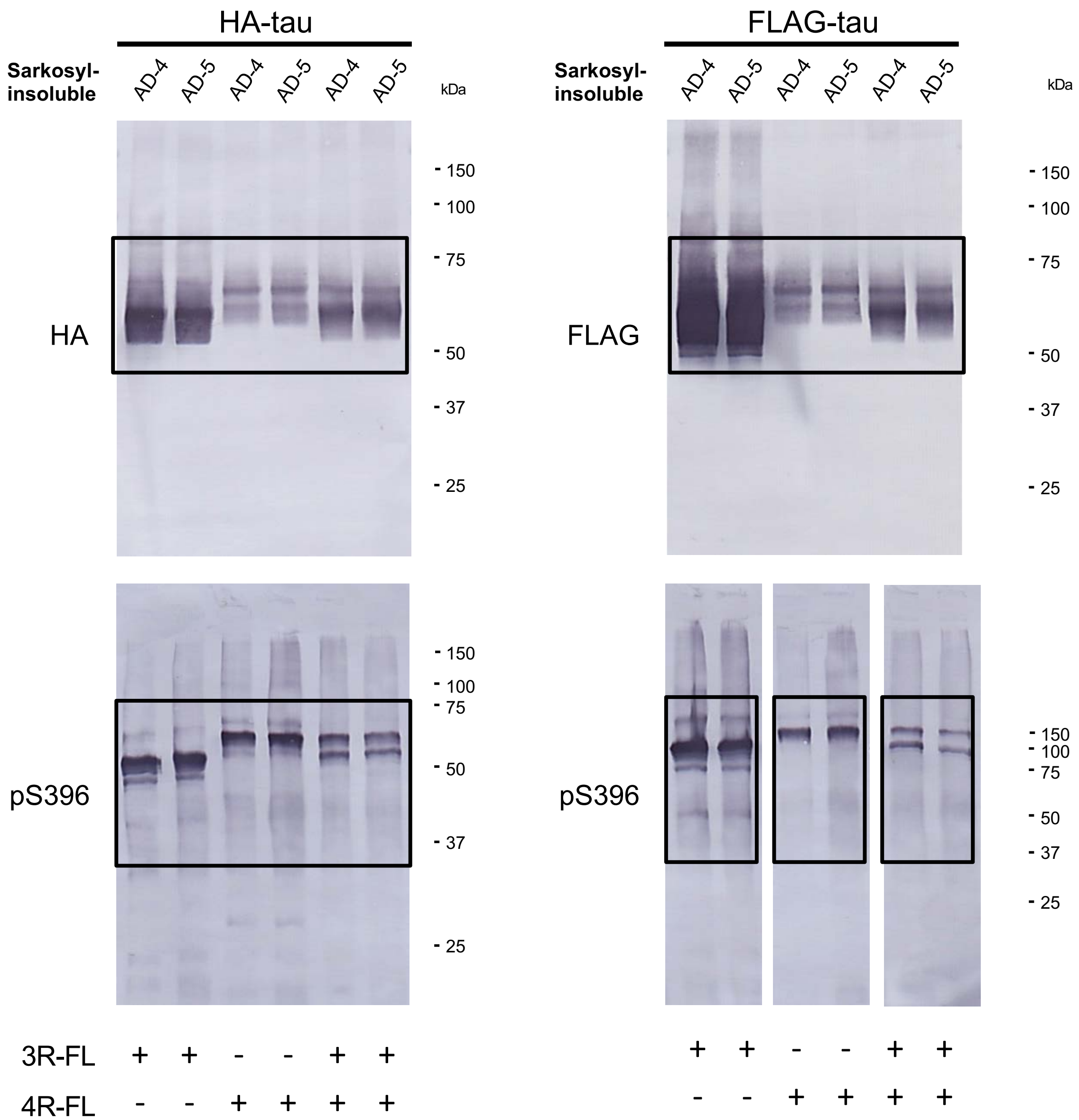

Source images of immunoblots

Supplemental Fig. 10A

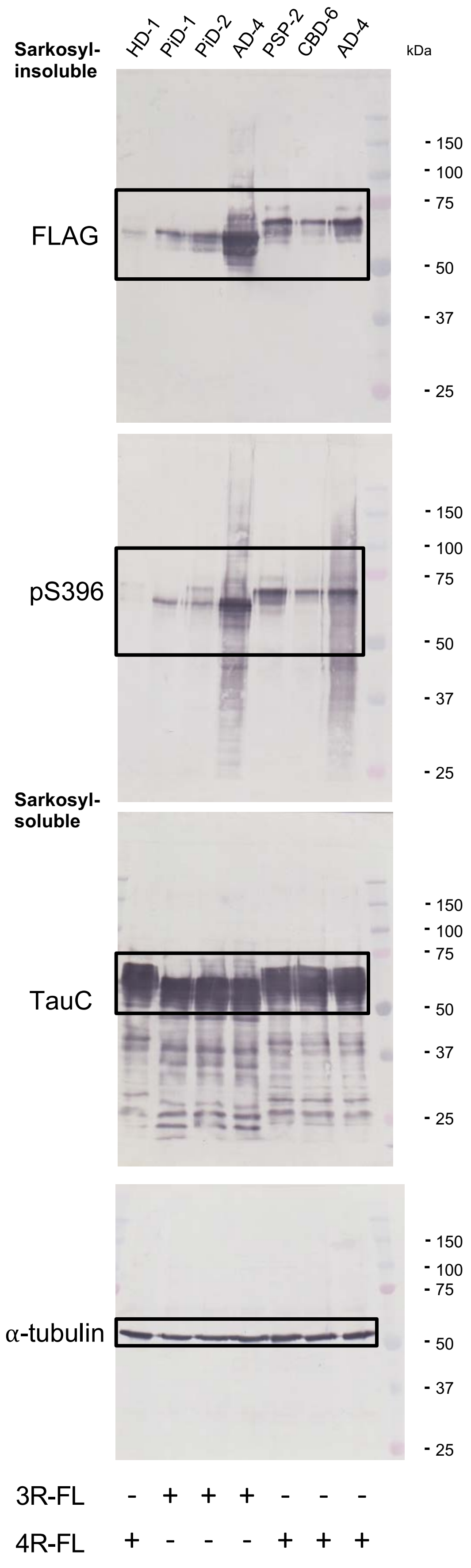

Supplement: awab091_Supplementary_Data [file awab091_supplementary_data.zip › awab091-suppl_data/brain-2020-01854-File011.pdf]
